# Josephson vortex loops in nanostructured Josephson junctions

**DOI:** 10.1038/s41598-018-21015-7

**Published:** 2018-02-09

**Authors:** G. R. Berdiyorov, M. V. Milošević, F. Kusmartsev, F. M. Peeters, S. Savel’ev

**Affiliations:** 1 Qatar Environment and Energy Research Institute, Hamad bin Khalifa University, Doha, Qatar; 2 Departement Fysica, Universiteit Antwerpen, Groenenborgerlaan 171, B-2020 Antwerpen, Belgium; 3 Department of Physics, Loughborough University, Leicestershire, LE11 3TU United Kingdom

## Abstract

Linked and knotted vortex loops have recently received a revival of interest. Such three-dimensional topological entities have been observed in both classical- and super-fluids, as well as in optical systems. In superconductors, they remained obscure due to their instability against collapse – unless supported by inhomogeneous magnetic field. Here we reveal a new kind of vortex matter in superconductors - the Josephson vortex loops - formed and stabilized in planar junctions or layered superconductors as a result of nontrivial cutting and recombination of Josephson vortices around the barriers for their motion. Engineering latter barriers opens broad perspectives on loop manipulation and control of other possible knotted/linked/entangled vortex topologies in nanostructured superconductors. In the context of Josephson devices proposed to date, the high-frequency excitations of the Josephson loops can be utilized in future design of powerful emitters, tunable filters and waveguides of high-frequency electromagnetic radiation, thereby pushing forward the much needed Terahertz technology.

## Introduction

Realizing linked and knotted excitations in space-filling fields is one of the key open questions in modern topology. To which extent an entire field can be twisted to allow formation of a loop or a knot is a highly nontrivial affair, requiring subtle interplay of topology and dynamics. Recent advances in fabrication and measurement techniques made it possible to realize such topological excitations in many areas of physics, including, for example, electromagnetism^1–3^, plasmas (see, for instance, ref.^4^ and references therein), liquid crystals^5–8^, and quantum^9,10^ and classical fluids^11–13^. Depending on the properties of the system, the knotted structures can be either static, as in the case of optical fields^1^, or disintegrate through a series of reconnections observed in fluids^11^. Superconductors, where elementary topological entities are the vortex lines of quantized magnetic flux, belong to the latter category. Namely, formation of vortex loops in superconductors is topologically allowed, but they have inherent energetic tendency towards annihilation. However, vortices in superconductors show much richer behavior compared to their classical counterparts in fluids^14,15^, which opens a broad exploration avenue for the physics of vortex loops. For example, in a thermally driven regime vortices transit into a liquid phase forming closed loop structures^16^. Thermal fluctuations and Berezinskii-Kosterlitz-Thouless physics are beneficial for the appearance of vortex loops in layered superconductors^17,18^. The filamentary nature of vortices enables vortex entanglement^19,20^ and vortex cutting and cross-joining processes^21^ due to, for example, vortex-vortex collisions or interactions with boundaries/defects or surfaces^22^, which can all lead to formation of knotted or linked vortex loops. Strong magnetic inclusions inside the superconductor can nucleate vortex loops that mimic the shape of magnetic field lines^23^. Nevertheless, although a number of theoretical works have addressed the vortex loop formation, vortex cutting and recombination processes over the years^21,23–26^, no distinct experimental signature of linked, knotted or isolated vortex loops has been found to date^27,28^.

**Figure 1:**
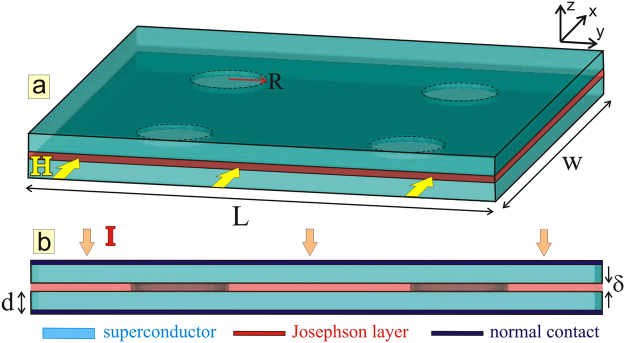
Schematics of the system. The perspective view (**a**) and the side view (**b**) of two superconducting layers (with rectangular planar area *L* × *w* and thickness $d\ll \lambda$) separated by a normal metal junction (thickness $\delta \ll \lambda$) in the presence of an in-plane field *H* and perpendicular current *I* (applied uniformly over normal contacts). Pillars (radius *R*), also superconducting, connect the two layers and form barriers for motion of Josephson vortices in the junction.

One of the reasons for elusive observation of vortex loops is that most research efforts were directed to Abrikosov vortex loops, which are typically very small, with radius of about superconducting coherence length *ξ* (see, e.g., ref.^21^), and difficult to stabilize for an extended period of time, both detrimental to their experimental verification. Interestingly, none of the earlier considerations dealt with Josephson vortices, which are arguably the most intriguing and most dynamic topological defects in superconductivity^29–32^, formed in junctions between superconductors, and quite essential to the layered high-temperature (high-*T*_*c*_) superconducting materials due to their prospects for THz technology^33^. In contrast to Abrikosov vortex loops^21^, Josephson vortex loops have no core and are far less constrained by coherence length^34^, thus can be much larger and can offer alternative ways of manipulation and stabilization in Josephson junctions with specially designed pinning sites. However, the available studies in the literature were restricted to 1D and 2D models of Josephson junctions, none of which considered three-dimensional interaction of Josephson vortices with a nanoengineered barrier, or any related phenomenon in a nanostructured Josephson junction. This was the exact objective of the study presented in this article, where we consider a three-dimensional Josephson junction of two superconducting layers separated by a normal metal (see Fig. 1), with an array of superconducting pillars inside the junction which serve as local barriers for the in-plane motion of Josephson vortices (JVs). Such geometry is readily realizable in experiment, and our results can easily be extrapolated to stacks of Josephson junctions or bulk high-*T*_*c*_ materials which are periodically perforated with holes subsequently filled with another superconducting material. Alternatively, granular superconductors with naturally attributed distribution of Josephson junctions and superconducting shortcuts can serve as a system where loops can be formed via the dynamical mechanism described in this article.

As a main result, here we reveal a novel topological entity in superconductors - the Josephson vortex loop. Our numerical simulations show that Josephson loops form around nanoengineered barriers as a result of cutting and recombination of regular Josephson vortices, as they circumvent barriers during their motion under the biasing current. We demonstrate that Josephson loops can remain stable in the system within a significant range of applied currents, as well as after all external drives are switched off. If applied current is large, Josephson loops undergo various scenarios of collapse, which are not only phenomenologically rich, but also leave clear transport signatures (in measured voltage for example), which can be used as a proof of existence of Josephson loops in the first place. As we discuss, Josephson loops can also be directly detected and studied using several available experimental techniques (ranging from 2D scanning-probe imaging to 3D-sensitive muon-spin rotation and small-angle neutron scattering measurements). Here reported peculiar responsiveness of the loops to applied magnetic field, current, local heating, as well as their characteristic dynamics in a tunable range of frequencies, make Josephson loops very relevant for the superconducting THz technology proposed to date (including more powerful THz emitters and new designs for THz photonic crystals and filters).

## Results

### Formation of Josephson vortex loops

As a representative example, we consider two superconducting layers of dimensions *L* × *w* × *d* = 100*ξ* × 100*ξ* × 5*ξ* (*ξ* being the coherence length at the working temperature) separated by a normal metallic junction of thickness *δ* = 1*ξ*, but connected by four pillars of radius *R* = 9*ξ* which represent barriers for the moving Josephson vortices (JVs) inside the Josephson junction formed between the top and bottom superconducting layer (see Fig. 1, and the Methods section for details of our Ginzburg-Landau simulations). The current is applied uniformly on the layers, namely perpendicularly to the top surface of the upper layer, and uniformly removed from the bottom surface of the bottom layer. In order to prevent the nucleation of Josephson vortex-antivortex pairs^35^ either at the edges of the sample or inside the system (depending on the state of the junction, see, for example, ref.^36^), we apply magnetic field parallel to the junction. Although with magnitude of just ~1% of the upper critical field *H*_*c*2_, the applied field is sufficient to induce Josephson vortices in the junction. Note that field *H*_*m*_ induced by superconducting (Meissner) currents in the sample is negligible compared to the applied field, for large ratio of magnetic penetration depth *λ* and the coherence length *ξ* (which is the case in experimentally fabricated junctions of Nb, NbN ($\lambda /\xi \sim 20$) or YBCO (*λ*/*ξ* > 100). The maximum of *H*_*m*_ can be roughly estimated as a product of the characteristic current density (always lower than the depairing current density ${j}_{dp}\approx \mathrm{2/3}\sqrt{3}{H}_{c2}\xi /{\lambda }^{2}$) and the characteristic size (coherence length) of strong currents flow near sample edges, resulting in ${H}_{m} < \mathrm{2/3}\sqrt{3}({\xi }^{2}/{\lambda }^{2}){H}_{c2}$ of the order 10^−4^*H*_*c*2_ for most junctions. This indicates that we can safely ignore any demagnetization effects in the rest of our analysis, since the fields generated by the screening currents are about 1% of the applied magnetic field (see also Supplementary Note 1 and Supplementary Fig. 1).

**Figure 2:**
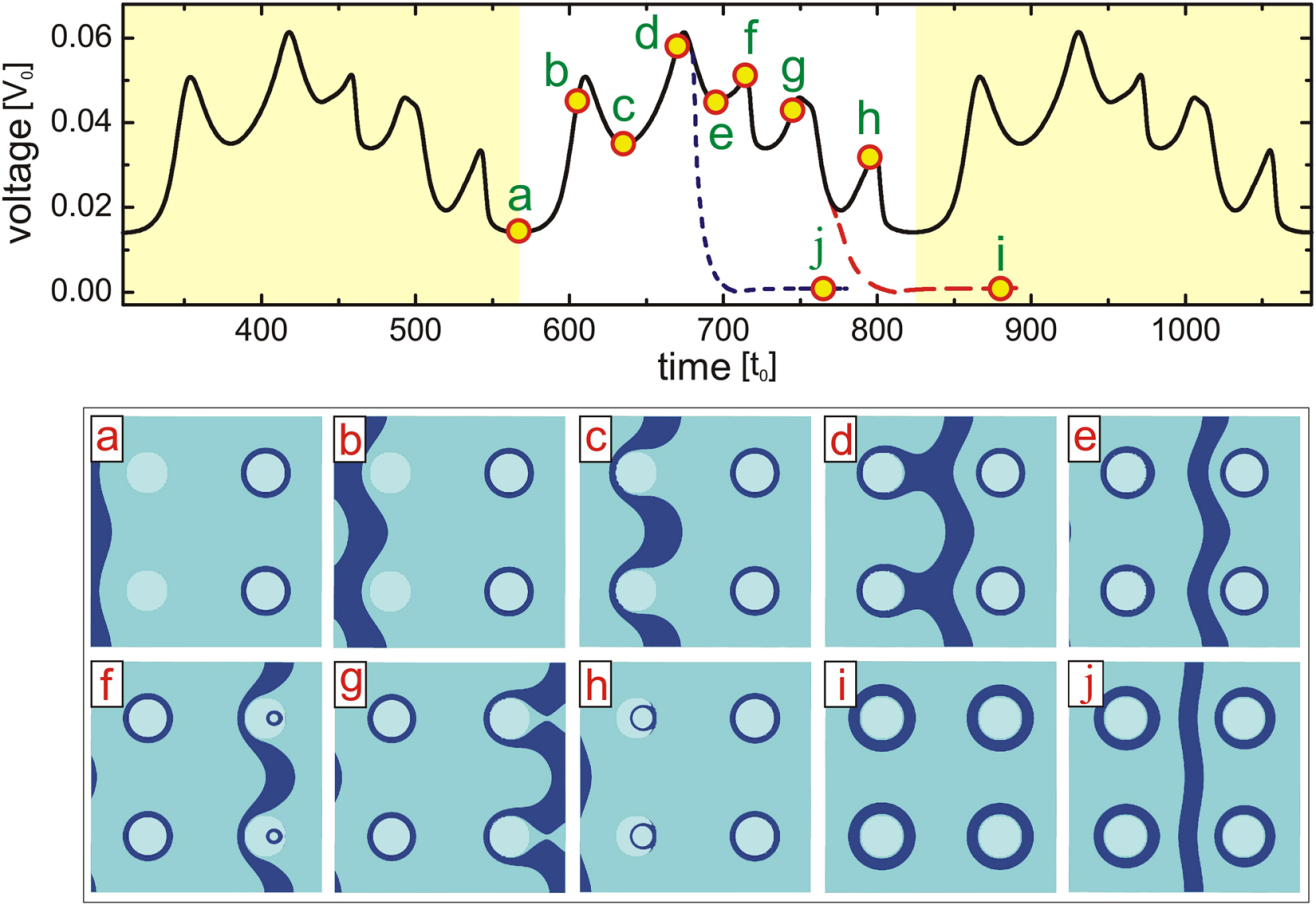
Formation of Josephson loops around the barriers in the junction through vortex cutting and recombination. Voltage-time characteristics of the sample with *L* = 100*ξ*, *w* = 100*ξ*, *d* = 5*ξ*, *δ* = 1*ξ*, and radius of the pillars *R* = 9*ξ*, for in-plane magnetic field *H* = 0.01*H*_*c*2_ and applied current density *j* = 0.055*j*_0_ (applied at *t* = 0), about 15% of the depairing current density *j*_*dp*_ = 0.38*j*_0_. For definition of all units we refer to the Methods section and Table 1. For this uniformly applied current density on the leads the distributed current density in the sample exceeds the critical Josephson current density *j*_*c*_ locally in the junction and the deparing current density locally inside in the pillars, which enables the onset of continuous flux flow. For here taken Cooper-pair mass and normal-state resistivity in the junction (*μ* = 1, *ζ* = 1, see Methods), we find *j*_*c*_ = 0.065*j*_0_. Dashed (red) and dotted (blue) curves show the voltage response of the system when the applied current was switched off at times *t* = 770*t*_0_ and *t* = 680*t*_0_, respectively. Panels (a–j) Show the isosurface plots of the Cooper-pair density at the times indicated in the main panel (taken isovalue is 30% of $|\psi {|}_{max}^{2}$, such that dark blue color outlines the vortex lines; pillars are shown by the lightest color). To visualize the vortex dynamics, please see the Supplementary Video 1.

Figure 2 shows the voltage-time [*V*(*t*)] characteristics of our sample, together with the evolution of the vortex state shown by isosurface plots of the Cooper-pair density, for an in-plane field of just 1% of the upper critical field and sufficient applied current to onset the flux flow in the junction. One can see from these plots that the dissipation arises from the periodic nucleation and motion of JVs. The minimum of *V*(*t*) at point *a* shown in Fig. 2 occurs due to the slow JV motion when overcoming the edge barrier to enter the junction. Acceleration of JV after passing the barrier results in a jump in the voltage curve (point *b*). A local minimum is observed in the *V*(*t*) curve when the JV slows down after reaching the nanostructured barriers (state *c*). The Lorentz force due to the applied current further drives the vortex across the barriers and results in a large deformation of the JV (see panel *c*). With time, the vortex finally engulfs the pillars, and accelerates after leaving behind the closed Josephson loops around the pillars (see state *d* and maximum *V*(*t*) near point *d*). The next minimum in the voltage curve is observed when the JV is temporarily trapped between the pillars (state *e*). The JV increases the pressure on the preexisting vortex loops at forthcoming barriers, until those penetrate the barrier area and collapse (shown in panel *f*) resulting in a local maximum in the voltage curve (see point *f*). With time the JV exits the sample after recreating closed loops around the pillars on its way (panel *g*). Note that once loops are formed, they remain under continuous pressure of the Lorentz force towards shrinkage, since opposite sides of the loop experience a Lorentz force in opposite directions. As a consequence, the formed loops may collapse inside the barrier area (panel *h*), which leaves as a trace a maximum in the voltage across the sample (see point *h*). After the collapse of the loop, a new JV enters the junction area and the entire process is periodically repeated, resulting in a periodic voltage response of the sample (see Supplementary Video 1).

Clearly, observation and further analysis of Josephson vortex loops would be facilitated if they were first stabilized as long-living entities. Here we demonstrate that Josephson vortex loops can indeed remain stable inside the sample after switching off the applied current, continuing the scenario shown in Fig. 2. Dashed red curve in Fig. 2 shows the voltage response of the system when the current was switched off after the JV has left the sample, leaving enclosed vortex loops around each of the pillars (state *g*). The system subsequently relaxes to the equilibrium state consisting of only “pinned” Josephson loops (see panel *i*). Dotted blue curve shows the time evolution of the voltage after the current was switched off when the JV was located between the pillars (after state *d*). In this case we remain with vortex loops around the pillars and a JV trapped between them (panel *j*). It is therefore likely that in a large multilayer system, or a bulk layered superconductor, the stationary state after switching off the current (and field) would comprise both Josephson loops and Josephson vortices.

### Josephson nature of the formed vortex loops

**Figure 3:**
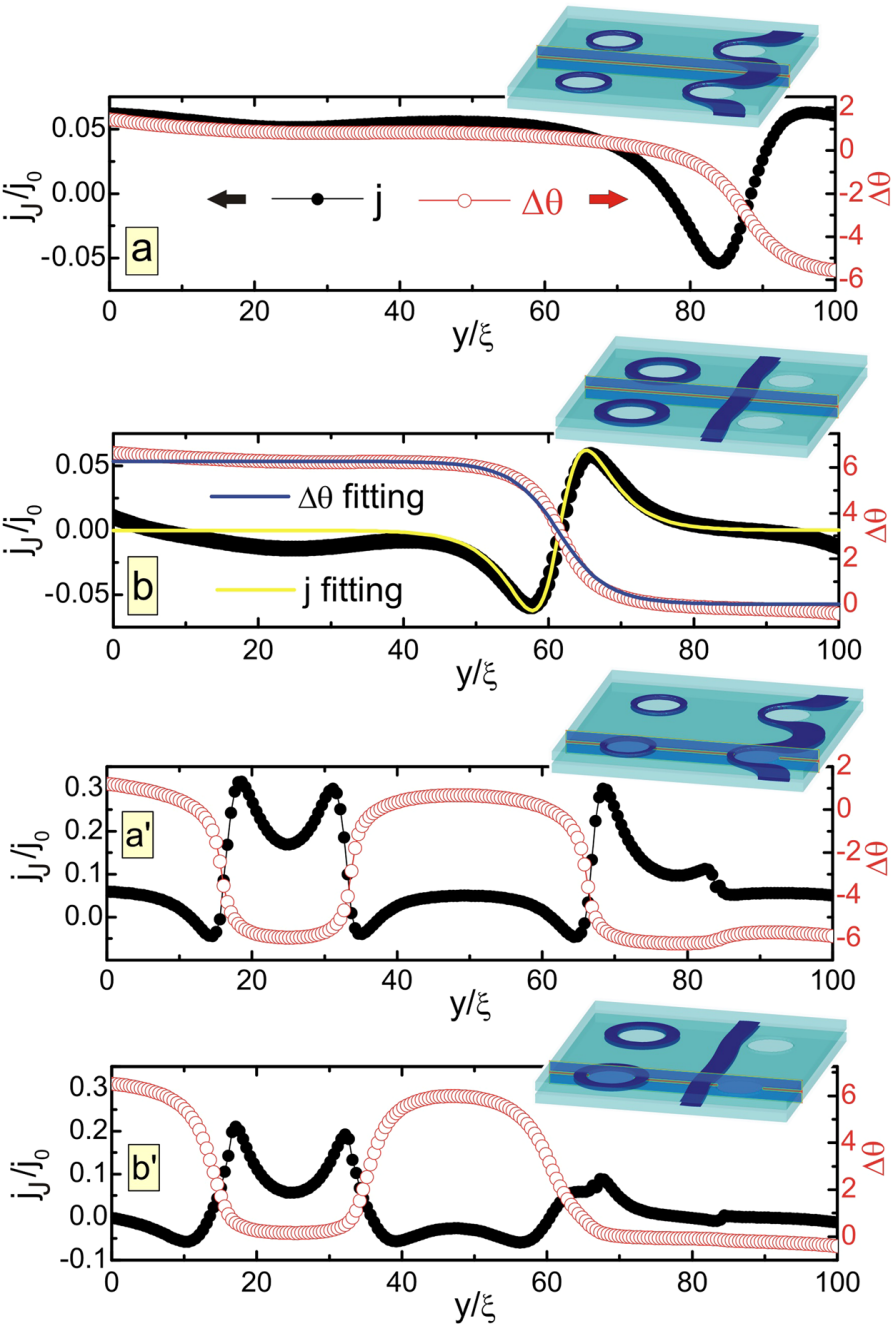
Josephson current and phase transfer through the junction. Panel (a) For the sample of Fig. 2 and a snapshot state *g* under applied current as in Fig. 2, we show the Josephson current density *j*_*J*_ and the gauge-invariant phase difference Δ*θ*, plotted along the junction in the cross-sectional plane depicted in the inset showing the corresponding isosurface plot of the Cooper-pair density). Panel (b) The same as in panel (a) but after the current has been switched off. The blue and the yellow curves are the obtained fits for Δ*θ* and *j*_*J*_, respectively, using standard Josephson relations (see text). Panels (*a*′ and *b*′) The same as in panels (a and b), but for the cross-sectional plane crossing the pillars (see insets). The observed 2*π* phase changes in Δ*θ* across the arms of the loop prove the Josephson nature of the looped vortex.

Although the isosurface plots in Fig. 2 are quite self-explanatory, we should properly characterize the found novel vortex matter from the point of view of other relevant quantities, particularly Josephson current and the phase across the junction. One expects the usual Josephson relation between the superconducting current and the gauge-invariant phase difference Δ*θ* between the top and the bottom superconducting layer of the junction. To demonstrate the consistency of our simulations with the standard Josephson relations, we considered the vortex state shown in Fig. 3, under an applied current (panels *a* and *a*′) and after the current was switched off (panels *b* and *b*′). Panels *a* and *b* correspond to the cross-section of the junction away from pillars, and capture just one Josephson vortex (the corresponding isosurface plots of the Cooper-pair density are shown as insets in Fig. 3). We plotted the gauge-invariant phase difference ${\rm{\Delta }}\theta ={\theta }_{{\rm{top}}}-{\theta }_{{\rm{bot}}}-\delta \bar{A}$ along the junction, where *θ* is the phase of the order parameter calculated in our simulations and indices “top” and “bot” refer to the bottom of the top layer and the top of the bottom layer, respectively, while $\bar{A}$ is the cumulative vector potential across the junction, $\bar{A}\equiv \mathrm{(1/}\delta )\,{\int }_{d}^{d+\delta }\,{A}_{z}dz$ (see Methods). A clear 2*π* change of the phase difference Δ*θ* is seen in these plots (red dots) due to the presence of the vortex. Black dots in panels *a* and *b* show the calculated Josephson current *j*_*J*_, which is somewhat distorted by the applied current (*a*). However, for current switched off (*b*), the phase difference and the current in the junction can be fitted by the standard Josephson vortex relations: Δ*θ* = 4 arctan(exp[(*y*_0_ − *y*)/*λ*_*J*_]) and *j*_*JV*_ = *j*_*c*_ sin{4 arctan[exp((*y*_0_ − *y*)/*λ*_*J*_)]}. Blue and yellow curves in Fig. 3b show results of such fitting, for vortex location *y*_0_ = 61.5*ξ*, Josephson penetration depth *λ*_*J*_ = 4.2*ξ*, and Josephson critical current density *j*_*c*_ = 0.0622*j*_0_, very close to numerically found value *j*_*c*_ ≈ 0.065*j*_0_. Small deviation of the fitting curves from the simulation results is due to the Meissner currents, which are not taken into account in the fitting expressions. Panels *a*′ and *b*′ in Fig. 3 show the results for the cross-section of the junction that crosses the pillars, hence capture one Josephson loop in addition to the Josephson vortex of panels *a* and *b*. A phase drop by 2*π* at the Josephson vortex is observed even in this cross section although the Josephson current is strongly affected by the presence of pillars, resulting in substantial spatial variation of *j*_*c*_ near the pillar. The change of 2*π* in Δ*θ* on one side of the pillar, followed by −2*π* change on the other, is a direct verification of the Josephson nature of the formed vortex loop.

### Mechanism behind vortex loop formation

To understand the mechanism of the loop formation around the pillars, one should consider two different situations when a vortex line approaches a barrier. If the external current is strong enough, the vortex cannot be stopped by the pillar - it either elongates and bends around the pillar, or crosses it transversely. In the first case, a loop has to be formed so that vortex can detach from the pillar, while for the second case the vortex passes the barrier without loop formation. Which mechanism wins is simply determined by the needed energy. Let *E*_1_ be the Josephson vortex energy per unit length and *E*_2_ the energy of a vortex per unit length inside the bulk superconductor. Then the energy needed for loop formation is about 2*πRE*_1_ while the energy required for vortex crossing through the pillar is close to 2*RE*_2_. Comparing these energies, one concludes that the loop can be formed if *πE*_1_ < *E*_2_.

To demonstrate this convincingly, we conducted additional simulations where we manipulated *E*_2_ by changing the critical temperature of the pillars. In the Ginzburg-Landau formalism, the variation of the critical temperature can be conveniently simulated through an expansion coefficient *α*(*T*) = *α*_0_(1 − *T*/*T*_*c*_) in the free energy functional of the system. Inhomogeneous *T*_*c*_(**r**) is then included in the calculation as *α*(**r**) = *α*(*T*)*f*(**r**) (see ref.^37^), where the spatially dependent thermal coefficient was taken to be *f*(**r**) = 1 in the superconducting layers and *f*(**r**) ≤ 1 inside the pillars. In real systems, such a suppression *f*(**r**) inside the pillar could correspond to lithographically pierced hole arrays in a high-*T*_*c*_ superconductor subsequently filled by a lower-*T*_*c*_ material, or locally heated pillars (made of the same material as bulk), by laser for example, resulting in spatial variation of temperature *T*(**r**).

**Figure 4:**
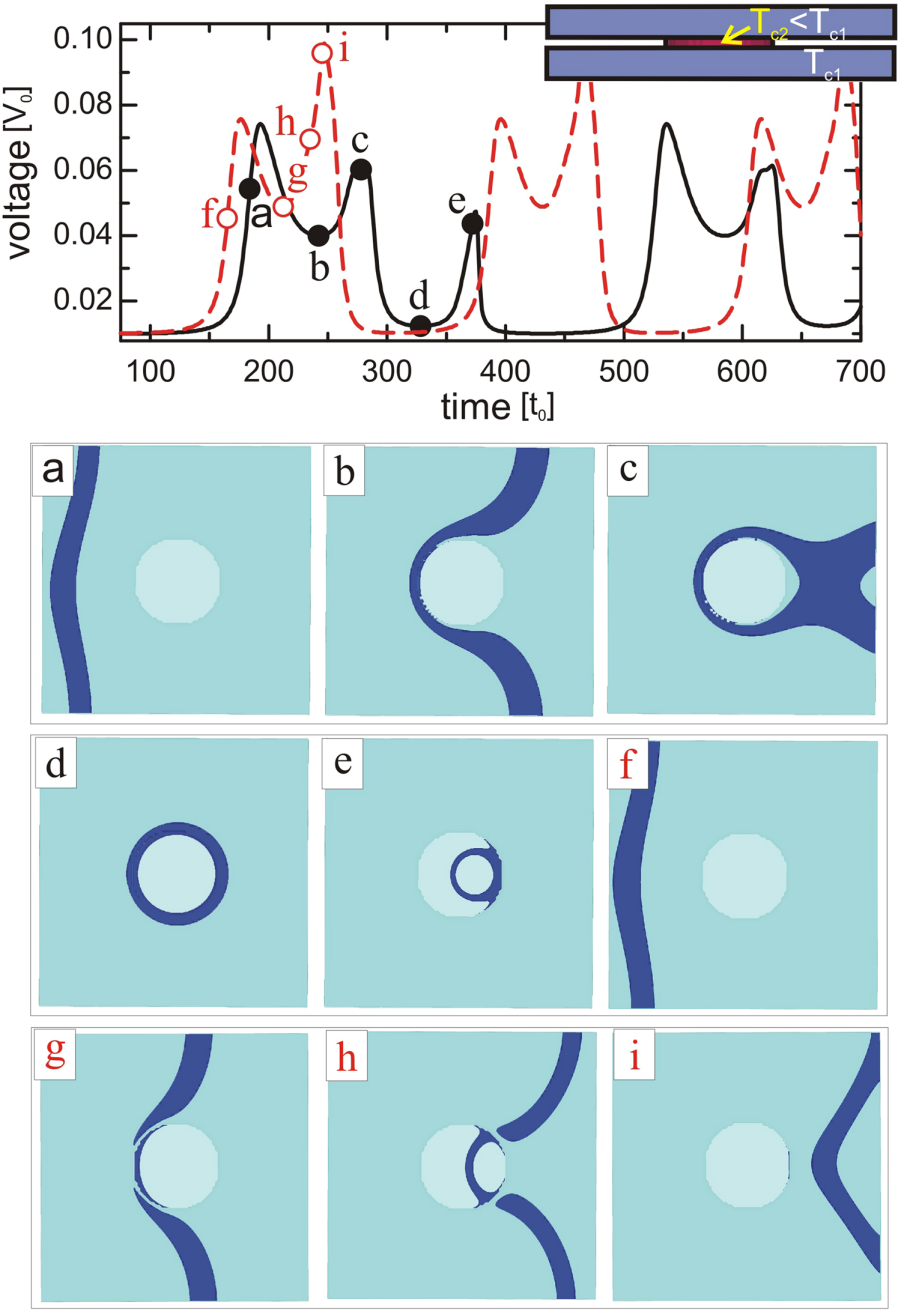
Barrier dependence of the loop formation. Temporal voltage response of the sample with *L* = 50*ξ*, *w* = 50*ξ*, *d* = 5*ξ*, *δ* = 1*ξ*, junction parameters *μ* = 1, *ζ* = 1, and one pillar of radius *R* = 8*ξ*, for the case of inhomogeneous *T*_*c*_ (modeled via spatial dependence of the coefficient *α*(**r**) = *α*(*T*)*f*(**r**), see text), where solid black curve corresponds to the homogeneous case (*f*(**r**) = 1 everywhere) and dashed red curve is the result for lowered *T*_*c*_ (*f*(**r**) = 0.25) inside the pillars. This can be reformulated by using the critical temperature *T*_*c*1_ outside and *T*_*c*2_ inside the pillars (see inset) and the relation *T*/*T*_*c*2_ = 1 − *f*(**r**) (1 − *T*/*T*_*c*1_). The applied current density is *j* = 0.047*j*_0_ and in-plane magnetic field is *H* = 0.02*H*_*c*2_. Panels (a–i) show the isosurface plots of the Cooper-pair density at times indicated in the *V*(*t*) curves (with color scheme as in Fig. 2).

Figure 4 shows the behavior of the sample with a single pillar inside the junction, for two different values of *f*(**r**) in the pillar (see sketch in the inset of Fig. 4). For the homogeneous situation (*f*(**r**) = 1 everywhere, that is the pillar and the superconducting layers are of the same material), the pillar represents a sufficient barrier for the vortex loop to be created (see panels a–d) though loop collapses under continued action of the applied current (panel e). However, once we sufficiently decrease the barrier by reducing the critical temperature of the pillar (taking *f*(**r**) = 0.25 inside the pillar) the loop is not formed and vortex penetrates the pillar (see panels f–i). These results clearly support our premise that the main mechanism for the formation of the loops in the here considered scenario is the interplay of energy needed for the deformation of the Josephson vortex and the energy barrier to penetrate the pillars. Here we should mention that we performed similar analysis for pillars up to 20*ξ* radius, and obtained Josephson loops without much effort. Since that required elongation of moving Josephson vortices by over 100*ξ*, we conclude that Josephson vortices exhibit unprecedented agility that by far surpasses one of Abrikosov vortices. As coreless objects, Josephson vortices were always expected to be more mobile, but we show here that their ability to cross, cut, twist, turn, deform or recombine is also superior to their Abrikosov counterparts.

We are very confident in this prediction, despite the known limitations of our theoretical model, considering the recent success of Ginzburg-Landau theory to describe experimentally observed Josephson vortices in mesoscopic geometries, even at very low temperatures^34^. We note that our theoretical approach becomes even more robust in samples with larger pillars, where the redistribution of current occurs on scales much larger than *ξ*, and both the order parameter and currents vary smoothly on the scale of *ξ*. For the samples with narrower pillars (below several *ξ*), the analysis within the non-linear 2D and 3D Usadel model^38^ would be valuable to support the proposed mechanism of loop formation.

### Current landscape for stabilization of Josephson loops

In what follows, we discuss the distribution of the current in the sample for applied current ranging from values insufficient to nucleate vortices in the sample to values that set vortices in continuous motion, which is intimately related to the mechanism of the loop formation. We begin with the current-voltage (I-V) characteristics, comparatively analyzed for the sample of interest, the sample without any pillars, and the sample with a pillar but with no Josephson coupling (*μ* = ∞). We intend to show that presence of pillars and consequent redistribution of applied current are instrumental for stabilization of Josephson loops in the junction. This comparative analysis requires extensive simulations at many different values of the applied current, hence for numerical convenience we consider a quarter of the sample considered in Fig. 2, namely a sample with dimensions *L* × *w* = 50*ξ* × 50*ξ* and just one pillar. Note that we first verified that the observed vortex behavior and the critical current densities are virtually the same as for the larger sample of Fig. 2.

**Figure 5:**
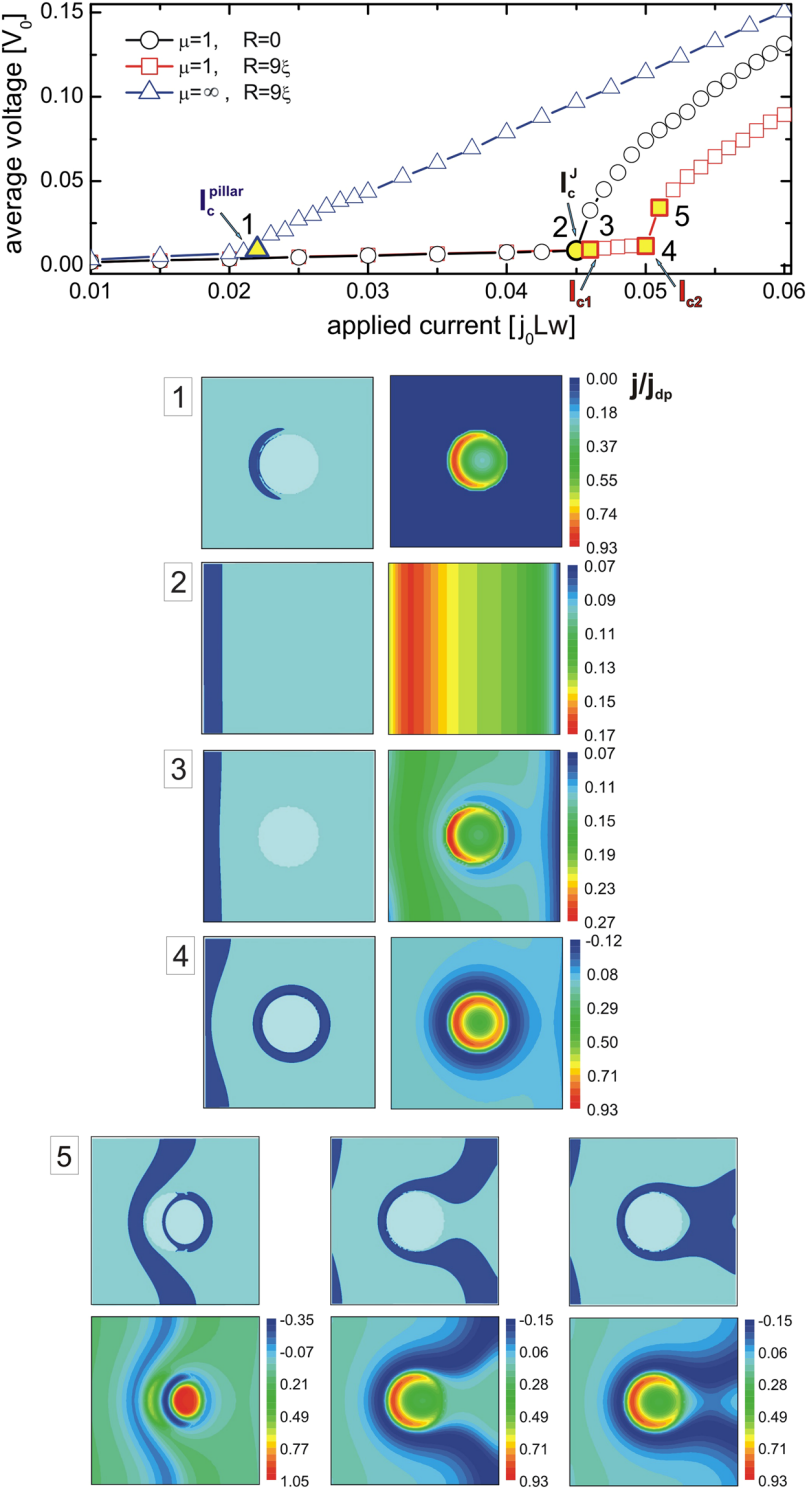
Current-voltage characteristics and current distribution related to vortex behavior. Main panel: Current-voltage (I-V) characteristics of the sample with *L* = *w* = 50*ξ*, *d* = 5*ξ*, *δ* = 1*ξ*, junction parameters *μ* = 1, *ζ* = 1, and applied magnetic field *H* = 0.01*H*_*c*2_: (i) with a pillar of radius *R* = 9*ξ* and no Josephson coupling (*μ* = ∞, blue triangles), (ii) with no pillar but with Josephson coupling (black circles), (iii) with both Josephson coupling and a pillar of radius *R* = 9*ξ* (red squares, closely corresponding to the case of Fig. 2). Labeling of the characteristic threshold currents in the main panel follows the detailed discussion in the text. Panels (1–4) Represent the isosurface plots of the Cooper-pair density and the corresponding current density distribution in the stationary states found at the applied currents indicated by yellow filled symbols in the main panel. Panel (5) same as panels 1–4, but shows the temporal sequence of non-equilibrium states upon onset of the resistive state in case (iii). Note: in panels (1–5) the current density is normalized by the depairing current density *j*_*dp*_ = 0.38*j*_0_.

The obtained I-V curves for the three considered cases are shown in the top panel of Fig. 5. The sample with pillar but without Josephson coupling exhibits the lowest critical current since all the applied current on the large top surface is focused through the narrow pillar. The resistive state is expected when the local current density at the pillar edge reaches the depairing current density *j*_*dp*_ = 0.38*j*_0_, which occurs at the critical current ${I}_{c}^{{\rm{pillar}}}=0.0225{j}_{0}Lw$ (see onset of resistance in the corresponding I-V curve). Just below ${I}_{c}^{{\rm{pillar}}}$, the local current density is very inhomogeneous and it nearly reaches the deparing current density at the edge of the pillar (about 93% *j*_*dp*_, see panel 1 of Fig. 5), even though the average current density in the pillar is ${\bar{j}}_{p}={I}_{c}^{{\rm{pillar}}}/\pi {R}^{2}\approx 0.2{j}_{0}$ (still only ≈53% of the deparing current density). For applied current above ${I}_{c}^{{\rm{pillar}}}$ the vortex periodically slips through the pillar, inducing resistive state and finite voltage without formation of any loops.

In the case of a plain Josephson junction (sample without any pillars) the onset to the resistive state occurs at the applied critical current ${I}_{c}^{J}\approx 0.045{j}_{0}Lw$, when the local current density near the edge (see panel 2 of Fig. 5) exceeds the maximal Josephson current density *j*_*c*_ ≈ 0.17*j*_*dp*_ = 0.065*j*_0_ (consistent with the value obtained from fitting the Josephson vortex in Fig. 3), and the Josephson vortex detaches from the edge and repeatedly crosses the junction, inducing the periodic oscillations in the voltage and a net nonzero resistance.

Having understood the fundamentals of its components, we now focus on our main system of interest, the two superconducting layers connected with the pillar and with Josephson coupling elsewhere. This system is not a simple superposition of the above two systems, and exhibits an intriguing dynamics with multiple threshold currents. At low applied currents, there are no loops nor vortices in the system, as the edge barrier prevents a Josephson vortex to penetrate the sample (panel 3 of Fig. 5). At the critical current for the loop formation *I* = *I*_*c*1_, the local current density at the edge reaches *j*_*c*_, the Josephson vortex is depinned from the sample edge and the system abruptly transits to a new stationary state with a *vortex loop enclosed around the pillar*. This transition is accompanied with considerable current redistribution in the system and current density at the junction edge drops below *j*_*c*_. We note also that the current density inside the pillar remains well below *j*_*dp*_, as can be seen in panel 3 of Fig. 5. With further increasing the applied current in the range *I*_*c*1_ < *I* < *I*_*c*2_, the Josephson loop remains stable. Panel 4 of Fig. 5 shows that at a current just below *I*_*c*2_ the current density at the pillar edge increases to about 93% of the depairing current density. In other words, at *I*_*c*2_ we observe the onset of the resistive state due to reached depairing current density in the pillar, as was the case also at ${I}_{c}^{{\rm{pillar}}}$ in the sample without Josephson coupling. However, in the system with Josephson coupling the loops are already formed for currents *I*_*c*1_ < *I* < *I*_*c*2_. Moreover, the resistive state in the latter case is much more complex, as already described in Fig. 2, with a Josephson loop collapse followed by Josephson vortex cutting and interconnection producing a new loop (see panel 5 of Fig. 5; the local current density in the pillar exceeds *j*_*dp*_ prior to collapse of the loop). We finally note that both *I*_*c*2_ and ${I}_{c}^{{\rm{pillar}}}$ can be tuned by the size of the pillar, but *I*_*c*2_ is always lower than ${I}_{c}^{J}+{I}_{c}^{{\rm{pillar}}}$, due to strongly nonlinear interaction of the current components flowing through the pillar and the rest of the junction.

After this analysis of the current density distributions, we stress again that although in our simulations the current is applied uniformly over the junction, it is subsequently redistributed (both inside the junction and across the superconducting layers) and is always larger inside the superconducting pillars than in the rest of the junction (see Supplementary Note 2 and Supplementary Fig. 2 for the vertical cross-sections and further discussion of the current distribution). This does not alter our described scenario for the formation of Josephson loops, but does bring to mind an alternative scenario - nucleation of Josephson loops from within the pillars, using large applied current only through the pillars. For completeness, we offer the full simulation of such a case in the Supplementary Note 3 and Supplementary Fig. 3, but we render it difficult to be realized experimentally. Namely, it would require spatial patterning of the current leads on top of pillars, then multiply larger applied current density compared to our original proposal for loop formation, and very robust superconductivity in the pillars to sustain such a large current and consequent heating, as well as to recover fast after current is switched off in order to stabilize Josephson loops against self-annihilation.

### Parametric phase diagram for Josephson loops

To start the discussion of possible experimental realization of our findings, we here provide a table of geometric and critical parameters corresponding to the sample of Fig. 2, assuming it made of Nb films. These parameters are within the experimental reach, especially since the recent progress in fabrication of small junctions reported in for example refs^39,40^. Note that, due to the used *μ* = 1 and the same normal-state resistivity of the junction ${\rho }_{n}^{J}$ as the bulk resistivity *ρ*_*n*_, the Josephson critical current of our junction, *j*_*c*_, is large and the junction resistance is low compared to nanofabricated junctions of refs^39,40^ (see Table 1 for a simulated sample made of Nb, with *j*_*c*_(*μ* = 1) ≈ 1.0 × 10^6^ A/cm^2^ and resistance-area product of ≈0.03 Ω*μ*m^2^). This motivated us to explore how vortex loop states evolve with lowering Josephson critical current *j*_*c*_(*μ*) $\propto$ 1/*μ* and increasing junction resistance ${\rho }_{n}^{J}={\rho }_{n}/\zeta$ (thus *ζ* is the ratio of bulk and junction resistivities) within a large range of parameters 1 < *μ* < 20 and 0.01 < *ζ* < 1. This brings the estimates for both the maximum Josephson critical current (*j*_*c*_(*μ* = 20) ≈ 5 × 10^4^ A/cm^2^) and the resistance-area product of the sample (up to ≈0.3 Ω*μ*m^2^) much closer to the experimentally realized values (respectively 10^4^–10^5^ A/cm^2^ and 1–10 Ω*μ*m^2^, see refs^39,40^). Note that in our samples the resistance-area product be conveniently estimated as $RA=2d{\rho }_{n}+\delta {\rho }_{n}^{J}=2d{\rho }_{n}\mathrm{(1}+\delta \mathrm{/2}d\zeta )$.

**Table 1:** The estimates of sample parameters for an experimental realization.

*A*	T [K]	L [*μ*m]	w [*μ*m]	d [nm]	*δ* [nm]	R [nm]
	4.2	1.6	1.6	80	16	150
	***j***_**0**_ **[A/cm** ^**2**^ **]**	***t***_**0**_ **[ps]**	***V***_**0**_ **[mV]**	***j***_***dp***_ **[A/cm** ^**2**^ **]**	***j***_***c***_ **[A/cm** ^**2**^ **]**	***RA*** **[Ω** ***μ*** **m** ^**2**^ **]**
	1.66 × 10^7^	0.67	0.49	6.31 × 10^6^	5 · 10^4^|_*μ*=20_ → 10^6^|_*μ*=1_	0.03|_*ζ*=1_ → 3|_*ζ*=0.01_
***B***	**T [K]**	**L [** ***μ*** **m]**	**w [** ***μ*** **m]**	**d [nm]**	***δ*** **[nm]**	**R [nm]**
	6.0	2.65	2.65	130	26.5	397.5
	***j***_**0**_ **[A/cm** ^**2**^ **]**	***t***_**0**_ **[ps]**	***V***_**0**_ **[mV]**	***j***_***dp***_ **[A/cm** ^**2**^ **]**	***j***_***c***_ **[A/cm** ^**2**^ **]**	***RA*** **[Ω** ***μ*** **m** ^**2**^ **]**
	3.65 × 10^6^	1.84	0.18	1.41 × 10^6^	1.1 · 10^4^|_*μ*=20_ → 2.2 · 10^5^|_*μ*=1_	0.05|_*ζ*=1_ → 5|_*ζ*=0.01_

(A) Geometric parameters of the sample shown in Fig. 1 and considered in Fig. 2, assuming it made of Nb films [with approximate parameters *ξ*(0) = 10 nm, *λ*(0) = 200 nm, critical temperature *T*_*c*_ = 7 K, and normal-state resistivity *ρ*_*n*_ = 18.7 *μ*Ωcm, and taking typical experimental working temperature of 4.2 K, so that *ξ*(4.2 K) ≈ 16 nm and *λ*(4.2 K) ≈ 320 nm in the Ginzburg-Landau model]. Bottom row gives the estimated values of units for current, time and voltage used in the calculations, the Josephson critical current density (depending on taken coupling in the junction *μ*), and the resistance-area product of the sample (where ${\rho }_{n}^{J}={\rho }_{n}/\zeta$). (B) *Idem*. as A., but for working temperature *T* = 6 K (so that *ξ*(6 K) ≈ 26.5 nm and *λ*(6 K) ≈ 530 nm, and all other quantities correspondingly rescaled).

The parameters presented in Table 1A are a guide for a corresponding experimental measurement on Nb-based samples, at *T* = 4.2 K. Do note however that our simulations are *temperature and material independent*, and can be adapted to any temperature or material provided that coherence length *ξ* and penetration depth *λ* are known at a given working temperature. To illustrate this, we provide in Table 1B the parameters of our samples if made of Nb, but at a different working temperature of *T* = 6 K (thus closer to critical temperature *T*_*c*_ = 7 K, supporting the validity of the Ginzburg-Landau model). At temperatures close to *T*_*c*_, one should consider the effect of fluctuations as well, which were omitted in the above calculations. Our analysis in Supplementary Note 4 and Supplementary Fig. 4 indicates that Josephson loops remain stable as long as inflicted fluctuations do not exceed 10% of the bulk order parameter.

**Figure 6:**
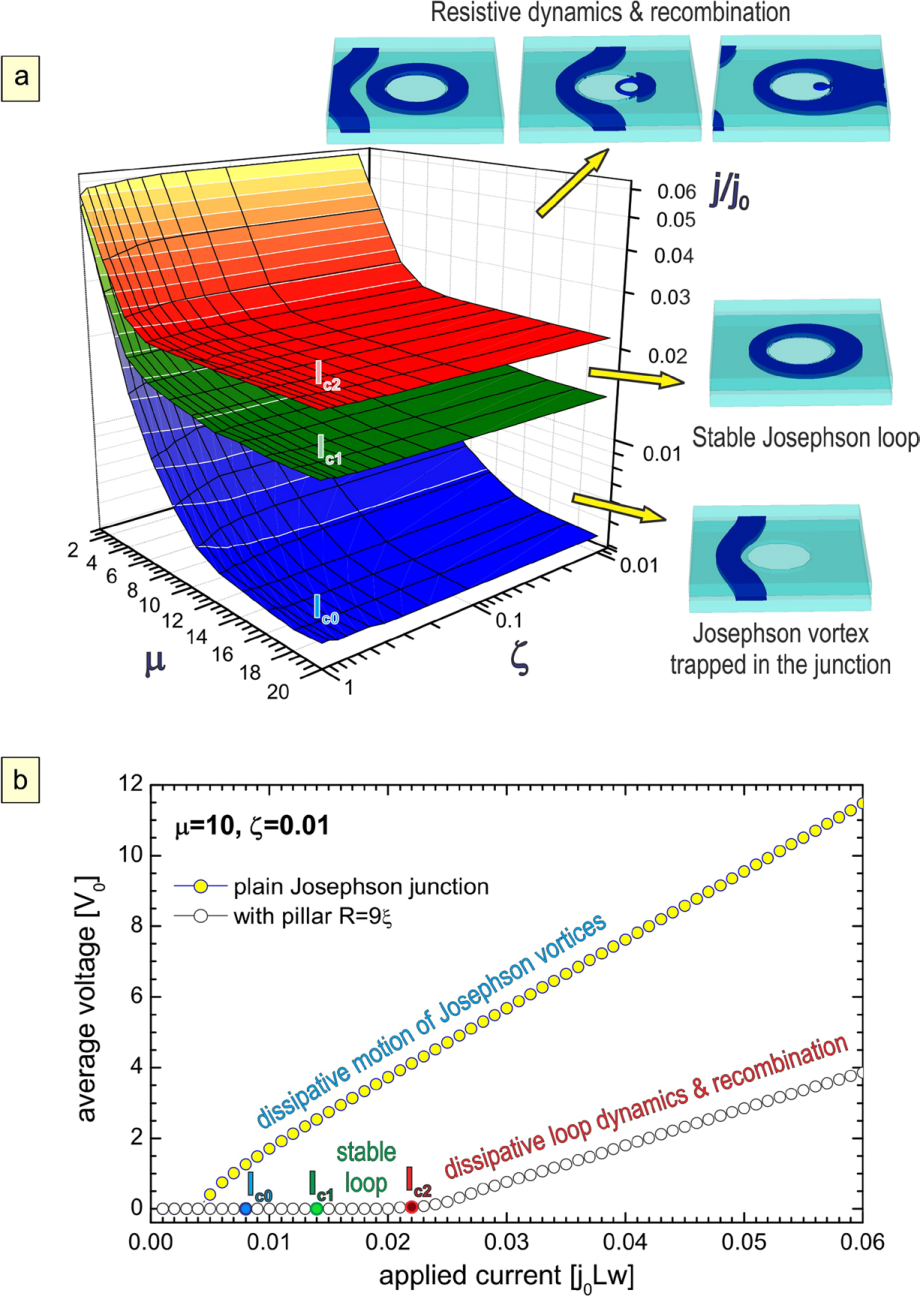
Josephson loop phase diagram as a function of the junction parameters. Panel (a) The critical currents for nucleation of the vortex in the junction (*I*_*c*0_), the formation of the Josephson loop (*I*_*c*1_), and the onset of flux flow (*I*_*c*2_), for the sample of Fig. 5 but for varied coupling (parameter *μ* = 1 − 20) and the normal state resistivity of the junction (${\rho }_{n}^{J}={\rho }_{n}/\zeta$, *ζ* = 0.01 − 1). Insets show exemplary states, obtained for *μ* = 10 and *ζ* = 0.01. Panel (b) Current-voltage (I-V) characteristics of the sample with *L* = *w* = 50*ξ*, *d* = 5*ξ*, *δ* = 1*ξ* and applied magnetic field *H* = 0.01*H*_*c*2_, compared for samples without a pillar and with a pillar of radius *R* = 9*ξ*, for the realistic case of relatively weak coupling and large resistivity in the junction (*μ* = 10, *ζ* = 0.01).

Exploring the broad range of parameters, we have identified different stable and dynamic Josephson vortex-Josephson loop states. Moreover, we detected one more threshold current, labeled *I*_*c*0_. Within the current range *I*_*c*0_ < *I* < *I*_*c*1_, the Josephson vortex, which has entered the sample, is trapped between the sample edge and the pillar. The loop is formed only at higher currents *I* > *I*_*c*1_ and remains stable until *I* = *I*_*c*2_. In Fig. 6(a), we show the parametric phase diagram, where three shown surfaces *I*_*c*0_(*μ*, *ζ*), *I*_*c*1_(*μ*, *ζ*) and *I*_*c*2_(*μ*, *ζ*) separate phases with no vortices, a Josephson vortex trapped between the edge and the pillar, the phase with a static Josephson loop, and the resistive state with moving Josephson vortices and successively creating and annihilating loops. All three threshold currents weakly depend on junction resistivity and decrease with increasing *μ* (lowering *j*_*c*_). However, the range of applied current [*I*_*c*1_, *I*_*c*2_] in which loops are stable broadens with increasing *μ* [compare current-voltage characteristics of Fig. 6(b) to the ones of Fig. 5; note also ≈10 times larger differential resistance in the dissipative state with dynamic loops, due to *ζ* = 0.01 in Fig. 6(b)]. This robustness of the state with a loop provides confidence that Josephson loops can be experimentally realized, even for parametric choices outside those considered in Fig. 6. Our results in all figures remain expressed in dimensionless units, to offer a general insight to experimentalists and a possibility to estimate the feasibility of the study for their particular experimental capabilities and samples. Namely, the units of our simulations (estimated in Table 1 for Nb) can vary strongly from sample to sample depending on disorder, properties of the junction (barrier materials, oxidation), junction homogeneity, alignment, and other sample properties that are challenging to control in the process of sample fabrication. In that respect, one should also consider various sources of microscopic vortex pinning in realistic samples, stemming from e.g. local variation of electronic mean-free path or critical temperature^41^. We did not include such pinning in the present analysis, assuming that its influence would be averaged out on the scale of the Josephson vortex core, hence would not affect the described dynamics of Josephson vortices and the reported formation of Josephson loops in our samples. Concerning the vortex dynamics prior to the formation of the loops, it may be influenced to a certain degree by pronounced inelastic scattering (short scattering time, see e.g.^42^), known to effectively “stiffen” the superconducting condensate and increase the viscosity for the vortex motion^43^. Still, such effects are far less relevant to Josephson vortices moving inside a superconducting junction.

### Experimental detection of Josephson vortex loops

All dynamic processes described in Fig. 2 leave traces in the voltage generated across the sample (as shown in Figs 2 and 4). These features are in the micro-to-milivolt range, and thus measurable. The loop formation, recombinations and collapse events are fast-occurring (in MHz-THz frequency range), but are definitely detectable in transport measurements. Note that the frequency of shown events depends on the applied magnetic field, current and temperature, and can be firmly tuned to the THz regime, especially in high-*T*_*c*_ superconductors where *ρ*_*n*_ is an order of magnitude larger than in Nb (see, for instance, ref.^44^ for YBCO) so that *t*_0_ $\propto$ 1/*ρ*_*n*_ of our calculations is correspondingly smaller and events faster. Thereby, the dynamic features associated with Josephson vortex loops are relevant to further design of THz technology, particularly *dc*-to-*THz* converters, THz emitters, but also filters/wavequides for electromagnetic radiation of frequencies matched by the (tunable) dynamics of the loops.

In a stationary state, we point out the possibility to directly visualize Josephson loops in applied tilted magnetic field, using a small out-of-plane component of the magnetic field to generate pancake vortices in the superconducting layers (see for example ref.^45^). Since there is a mutual attraction between pancake and Josephson vortices (as discussed in refs^45–52^ and references therein), pancake vortices are expected to “decorate” Josephson loops and form ring structures - visible by standard surface imaging techniques (including Scanning Hall-probe Microscopy^53^, Magnetic Force Microscopy^54,55^, Scanning Tunneling Microscopy^56,57^, and other), as has been demonstrated experimentally by several groups for the case of regular Josephson vortices^53,58–63^. Another possibility to directly detect Josephson loops is the muon-spin rotation (*μ*SR) measurement^64^, since muons will be scattered from the loops as a 3D magnetic object, and leave a clear signature of that - distinct from any other signature of quasi 2D objects such as Abrikosov, Josephson or pancake vortices^65^.

**Figure 7:**
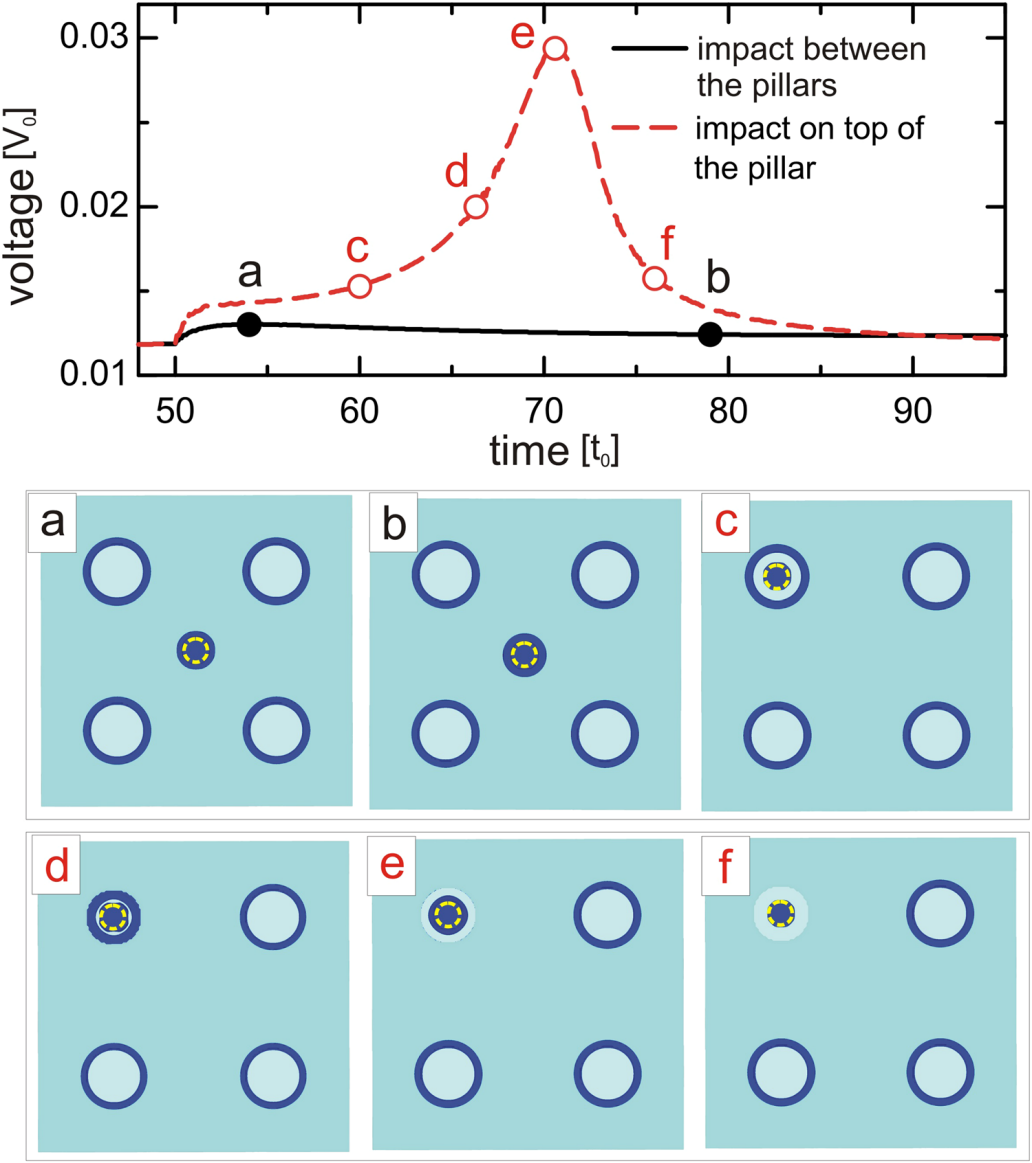
Response of the Josephson loop to a focused laser beam. Voltage-time response of the sample of Fig. 2, when the laser impact (at *t*_*l*_ = 50*t*_0_) occurs between the pillars (solid black curve) or on top of either pillar (dashed red curve). The applied current density is *j* = 0.047*j*_0_, in absence of magnetic field. Panels (a–f) Isosurface plots of the Cooper-pair density at times indicated in the *V*(*t*) curve (with color scheme as in Fig. 2). Dashed yellow circles denote the hot spot used to simulate the laser impact.

Moreover, some direct imaging techniques can benefit even from dynamic responsiveness of the Josephson vortex loops. Here we particularly have in mind the low-temperature Laser Scanning Microscopy (LTLSM)^66^, where imaging is made based on the voltage response due to the local motion and dissipation of vortices under action of the laser (local heating). This means that in our system, when laser acts away from the Josephson loops the voltage response will be minimal, whereas maximal voltage response is expected when the impact of the laser occurs near the loops, causing their shrinkage or expansion. We have tested this premise in the sample with the same parameters as in Fig. 2, where the vortex loops around the barriers were shown to remain stable when the applied drive is significantly reduced. In our Ginzburg-Landau simulation, the impact of the laser is taken into account as a local increase of the temperature (“hot spot”), using the spatially and time dependent *α*(*t*, **r**) = 0 inside the hot spot. In other words, we increase the local temperature to *T* = *T*_*c*_ for *t* > *t*_*l*_, where *t*_*l*_ is the moment of the laser action. Figure 7 shows the voltage response of the sample when the laser acts between the pillars (solid-black curve) or on top of any of the pillars (dashed-red curve). In the former case, very weak dissipation is observed due to the formation of a hot spot (panels a and b). However, when the laser acts near the loops (in this particular case on top of the pillar, see panel *c*), the annihilation of the vortex loop is observed (panels d–f), which results in a pronounced voltage peak across the sample. We therefore conclude that such dynamics can be distinctly detected by LTLSM.

Of course, the exact distribution of heat due to action of a laser on a particular sample, and the consequent behavior of the loops and the measured signal, can only be assessed if the time-dependent heat diffusion is considered on equal footing as the vortex motion. As discussed in more detail in ref.^67^, the heat capacity of the sample controls the speed of local heating/cooling, whereas the heat conductivity controls the spatial extent of heating. Vortex motion itself heats the sample and favors alignment of vortex trajectories^43,68^. Although those affect the dynamical processes in the junctions such as ours, we do not expect that exact description of local heating will change any of the fundamental mechanisms of Josephson loop formation and stabilization reported in this manuscript.

## Discussion

The present simulations have revealed several important features in the dynamics of Josephson vortices in (artificial) Josephson junctions with pillars or other nanostructured barriers, which can be used for the realization of Josephson vortex loops in superconducting systems. The observed dynamics is characterized by vortex cutting and subsequent recombination processes during circumvention of the barriers, resulting in stable closed loops around the barriers. The life time of these vortex loops, as well as their further dynamics, depend on both the external parameters (such as the applied current and magnetic field) and the parameters of the system itself (for example, the size of the barriers, the thickness of both superconducting and metallic layers). In what follows, we discuss several more novel and essentially non-adiabatic processes associated with the formation, existence, and destruction of Josephson loops.

**Figure 8:**
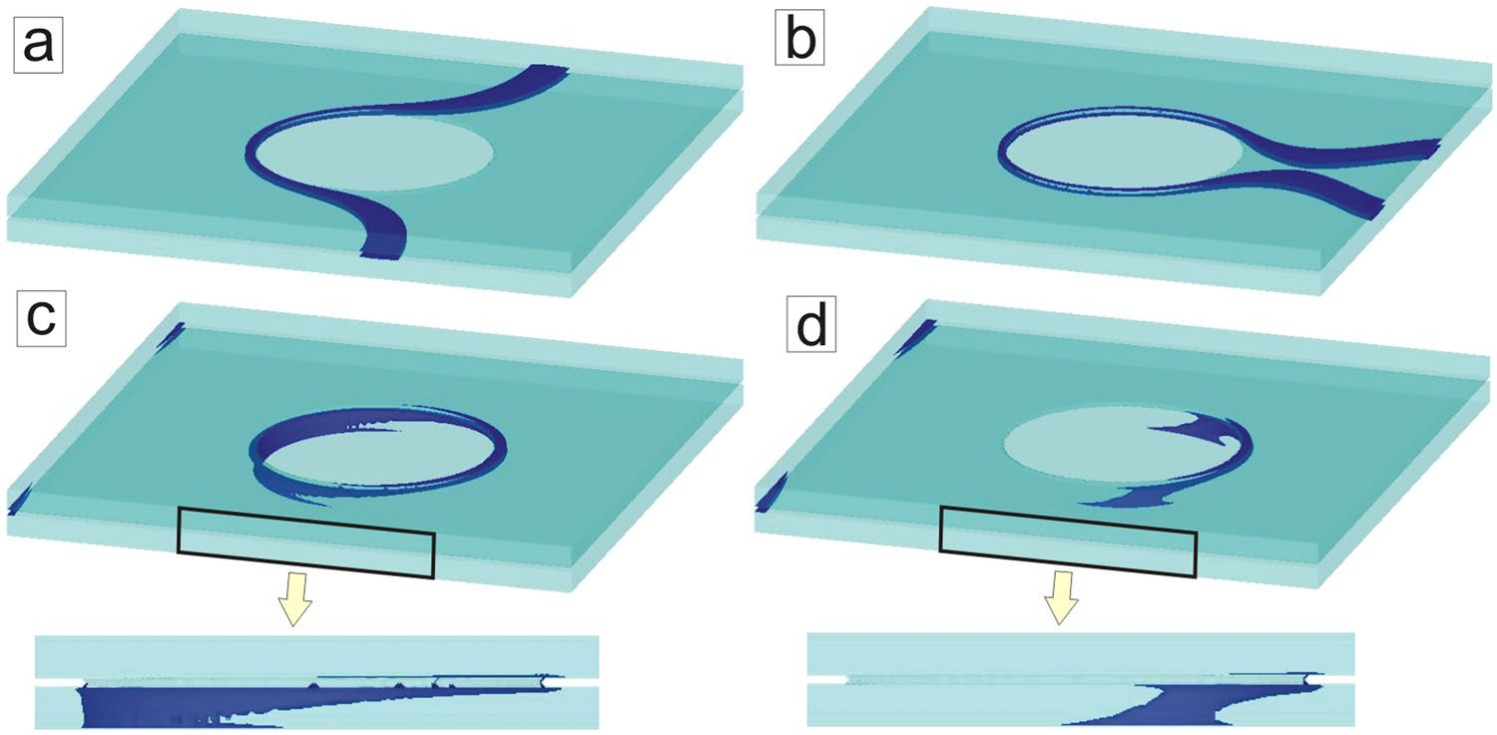
The escape of the Josephson vortex loop perpendicularly to the junction. Isosurface plots of the Cooper-pair density at time intervals *t* = 660*t*_0_ (**a**), *t* = 740*t*_0_ (**b**), *t* = 780*t*_0_ (**c**) and *t* = 790*t*_0_ (**d**) showing the dynamics of the Josephson vortex in the presence of a pillar of size *R* = 15*ξ*, formation of the loop around the pillar, and its tilt. The bottom row shows a zoom of the side view of (**c**,**d**). The applied current density was *j* = 0.055*j*_0_ (applied at *t* = 0) and the in-plane field *H* = 0.01*H*_*c*2_. Sample with *μ* = 1,*ζ* = 1 and dimensions are *L* = 100*ξ*, *w* = 100*ξ*, *d* = 5*ξ* and *δ* = 1*ξ*.

Obviously, the size of the pillars plays an important role in determining the temporal stability of the vortex loops and the mechanism through which they dissolve. For example, as was shown in Fig. 2, for the smaller size of the pillars the loops collapse inside the barrier area due to the Lorentz force of the applied current, which enforces the contraction of the loops. One can expect a different scenario for the case of a larger barrier, so that it prevents the penetration and collapse of the loops. To examine this situation, we show in Fig. 8 the dynamics of the Josephson vortex (JV) for a sample with a single pillar of larger size (*R* = 15*ξ*). As is intuitive, the JV has to deform strongly to circumvent such a large barrier [see Fig. 8(a)]. Nevertheless, even for this larger size of the barrier we observe the formation of an enclosed loop around a pillar [Fig. 8(b)]. This is in agreement with our qualitative adiabatic analytical condition of loop formation *E*_2_/*E*_1_ > *π*, which does not depend on the pillar radius, allowing loop formation around very wide pillars. However, due to the continued action of the current *I* > *I*_*c*2_, the loop should lose its stability. It turns out that penetration and collapse of the loop inside the barrier is energetically very costly, and therefore the loop prefers to tilt perpendicular to the junction and penetrate the superconducting layers, as shown in Fig. 8(c) (see also zoom-ins in the bottom row of Fig. 8). Note, this process breaks the symmetry of the initial problem in vertical direction. The loop breaks open when it reaches the surface of the sample (bottom side in this particular case) and leaves the system as a semi-loop which shrinks down to annihilation while avoiding the pillar [Fig. 8(d)]. Note that in the case of breaking vertical symmetry in a multilayer and more loops above/below the one we discussed in Fig. 8, the adjacent loops would simply tilt, open and shrink in parallel to each other, or form a vortex helix, the new superconducting vortex entity similar to magnetic helical structures.

**Figure 9:**
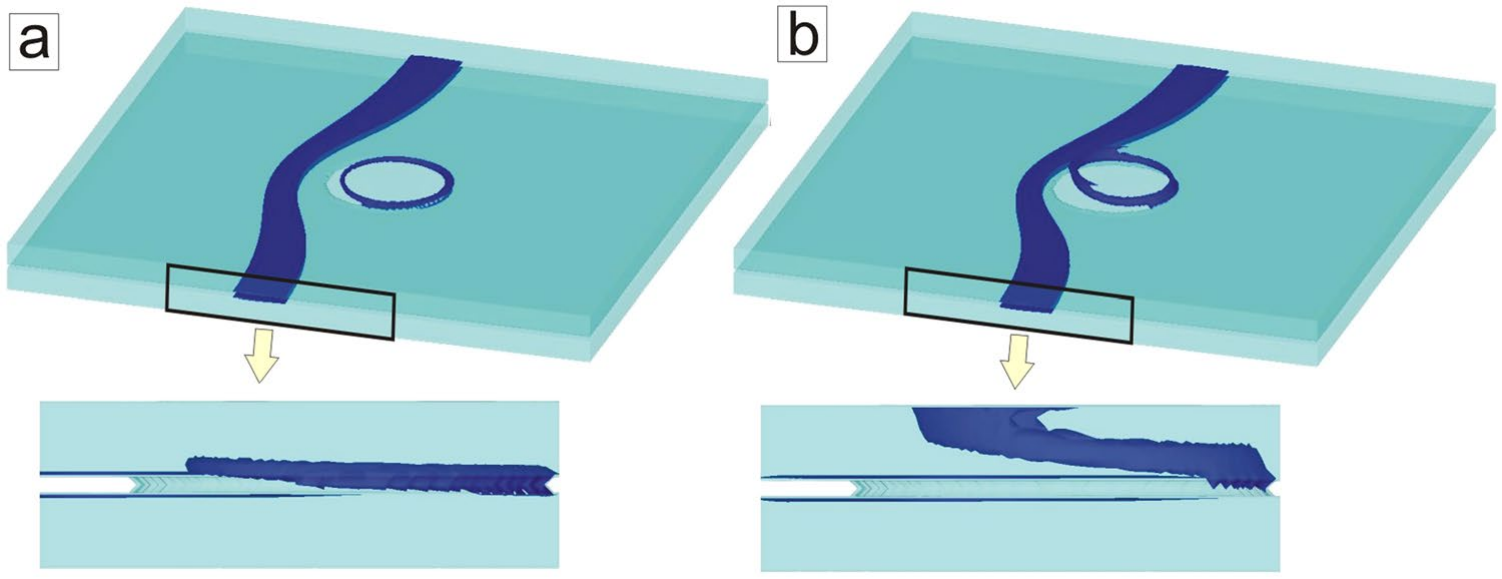
The escape of the Josephson loop under pressure from the incoming Josephson vortex. Isosurface plots of |*ψ*|^2^ (with time interval of *t* = 30*t*_0_) at *H* = 0.01*H*_*c*2_ and *j* = 0.057*j*_0_, for same sample parameters as in Fig. 2, but with just one pillar. Due to faster speed of the incoming Josephson vortex as compared to Fig. 2, the dynamic process of the collapse of the Josephson loop changes from shrinking inside the barrier to a perpendicular tilt and escape around the barrier.

The variation of the external parameters (for example the applied current and/or magnetic field) controls the lifetime of the loop and the mechanism of its collapse. This is illustrated in Fig. 9, where we plot the evolution of the enclosed Josephson loop when the subsequent JV approaches it. Due to their mutual repulsive interaction, the new JV pushes the loop in the direction perpendicular to the junction [see Fig. 9(a)]. The loop eventually reaches the top surface of the sample [Fig. 9(b)] and leaves the system in a previously described fashion. Note, however, that here the size of the pillar is *R* = 9*ξ*, where the Josephson loop on its own would collapse inside the barrier - as discussed in Fig. 2. In spite of that, and the fact that the current is larger in the present situation as compared to Fig. 2, such that we have a larger Lorentz force acting to collapse the loop, the increased speed of the incoming Josephson vortex completely changes the process of destruction of the loop. This supports our earlier statement about the variety of different stable and dynamic Josephson vortex-Josephson loop states when changing external and/or geometrical parameters. This illustrates the relevance of the timescales of the different involved dynamic processes and the fundamentally non-adiabatic character of the discussed phenomenon, going beyond our initial qualitative and adiabatic description of the loop formation mechanism via energy comparison, and worthy of further investigation.

Our simulations have also revealed one more mechanism for the destruction of Josephson loops in a nanoengineered junction, particularly fascinating since it involves the change in chirality of the loops. In this scenario, the incoming vortex erases the preexisting loop, and does not leave a new loop behind. Such a scenario is more likely to take place for smaller thickness of the superconducting layers, as is, for example, the case in bulk layered superconductors.

**Figure 10:**
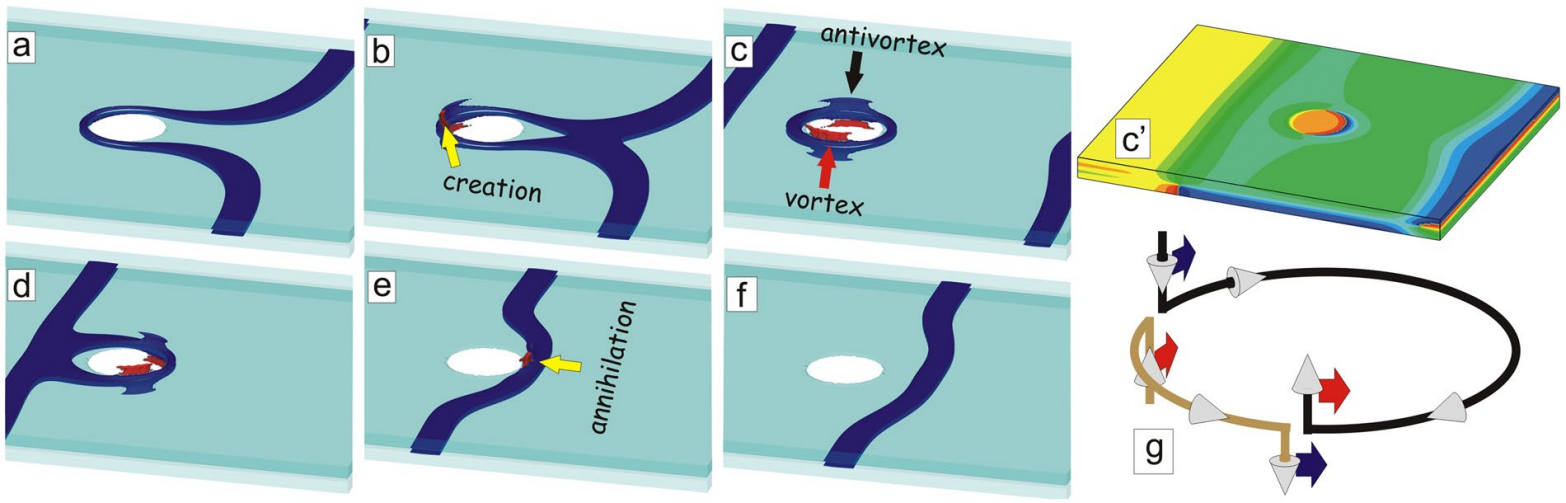
Change of chirality of the Josephson loop prior to annihilation with a moving Josephson vortex. Creation of the Josephson loop (**a**,**b**), its consecutive change of chirality (**c**,**d**), and annihilation with the incoming Josephson vortex (**e**,**f**), shown as isosurface plots of |*ψ*|^2^. The change of chirality occurs via creation and motion of vortex-antivortex pairs in the superconducting layers interconnected by semiloops in the junction area, as schematically shown in (**g**). The sample with *μ* = 1,*ζ* = 1 and dimensions are *L* = 120*ξ*, *w* = 100*ξ*, *d* = 4*ξ*, *δ* = 1*ξ* and the radius of the pillar is *R* = 8*ξ*. The magnetic field is *H* = 0.01*H*_*c*2_ and the current density is *j* = 0.052*j*_0_. The shown snapshots are taken at times *t* = 1080*t*_0_ (**a**), *t* = 1090*t*_0_ (**b**), *t* = 1115*t*_0_ (**c**), *t* = 1130*t*_0_ (**d**), *t* = 1060*t*_0_ (**e**) and *t* = 1180*t*_0_ (**f**) (*t* = 0 refers to switching on the current). Panel (c’) shows a contourplot of the phase of the order parameter at the surfaces of the sample at time *t* = 1115*t*_0_ [the state shown in (**c**)]. To visualize the vortex dynamics, please see Supplementary Videos 2 and 3.

To illustrate this mechanism, we show in Fig. 10 the topological evolution of moving JVs in the sample with slightly thinner layers than above (*d* = 4*ξ*). As one expects, the JV becomes strongly deformed near the barrier [Fig. 10(a)], creating an enclosed loop around the barrier [Fig. 10(b)]. Instead of observing the collapse of the loop in the barrier area, or its tilt towards the top/bottom, we now found that two Abrikosov vortex-antivortex (or “pancake-antipancake”) pairs are created in the top and bottom layer of the junction [highlighted by red/black arrows in Fig. 10(c), see also Fig. 10(c’)]. The top pair provides a topological solution for the loop to split open in the top layer, whereas the bottom pair interconnects by a new loop in the junction area, with opposite chirality to the previous one. Subsequently, both pairs traverse the perimeter of the barrier, annihilating on the other side, so that the first existing loop shrinks to annihilation, and the new loop is fully formed (see cartoon in Fig. 10). Due to its reversed chirality, the new loop now attracts the incoming JV, which results in their recombination [Fig. 10(d)] and easy “tunneling” of the Josephson vortex through the barrier [Fig. 10(e)] without leaving a Josephson loop behind [Fig. 10(f)]. Please find in Supplementary Video 2 our animated data of the evolution of the Cooper-pair density and the phase of the order parameter during this process.

In summary, we predict formation and stabilization of new 3D topological objects in superconductors - Josephson vortex loops - in Josephson junctions and layered superconductors with nanoengineered barriers for the motion of Josephson vortices under an electric drive. Josephson loops are then created via cutting and recombination of moving Josephson vortices, and remain stable in the sample in a range of applied currents as well as after the applied current is switched off. When critical to destabilize (depending on the size of the barriers, applied magnetic field and current, as well as other parameters of the exact system of interest), Josephson loops can either self-annihilate inside the barrier, or tilt perpendicularly to open and shrink to disappearance next to the barrier, or vertically interconnect with adjacent loops in the stack and form novel helical structures. The further incoming Josephson vortices can pressurize the loops to collapse, but then pass the barrier and create new loops instead of the collapsed ones. However, incoming Josephson vortices can also erase a preexisting loop, if the loop changes its chirality beforehand. The latter process is quite fascinating, as it involves a spontaneous shrinkage of one loop and the creation of another with opposite circulation, so that the barrier becomes transparent to the incoming Josephson vortex via recombination, in remote analogy to the Klein paradox^69^.

Josephson loops are therefore a very rich study object, detectable in experiment by direct imaging in tilted magnetic field, using muon-spin rotation, small-angle neutron scattering, laser scanning microscopy, or via traceable features in transport measurements. These loops are crucial for the understanding of the dynamics of Josephson vortices in the presence of a pinning landscape and may be a missing link towards realizing more complicated knotted and linked topological structures in superconducting systems, as well as new advanced emitters (exploiting radiation of moving Josephson vortices^70,71^), filters of THz radiation (so-called THz photonic crystals, see for example refs^72.73^), THz superconducting detectors based on Josephson plasma surface waves^74^, THz nonlinear and quantum devices^75,76^, as well as THz wave guides^77^. Generally speaking, Josephson loops will be relevant to any layered system with spatial inhomogeneities, should those be inclusions of any shape, or regions of higher Josephson coupling. Furthermore, the vortex loops can form in granular high-temperature superconductors and be linked to the experimentally observed paramagnetic Meissner effect^78,79^, since superconducting granules of arbitrary orientations and shapes can inter-penetrate and form superconducting shortcuts in the surrounding Josephson media leading to interconnected loops and the onset of spontaneous magnetization. Finally, we have also shown that Josephson loops can be created by local current injection, similarly to the observed stabilization of the vortex loops by the current flow in liquid Helium IV^80^. Exploring these analogies further is bound to lead to a better understanding and control of vortex loops in superconductors, as well as to reveal a plethora of novel phenomena.

## Methods

### Theoretical framework

Our model system consists of two superconducting layers separated by a normal metal junction, where an array of pillars (each of radius *R* and of either the same superconducting material as the layers or material with lower critical temperature) connects the superconducting layers (see Fig. 1). For this system, we solved the dimensionless time-dependent Ginzburg-Landau (GL) equation:

1 $$u\,(\partial /\partial t+i\phi )\,\psi ={(\nabla -i{\bf{A}})}^{2}\psi +(f({\bf{r}},t)-|\psi {|}^{2})\psi +\chi ({\bf{r}},t),$$

where *f*(**r**, *t*) accounts for spatially and/or temporally varied temperature (as for the case of switching local heating by laser) or critical temperature in the pillars if they are made of different superconducting material^37^. Fluctuating field *χ*(**r**, *t*) allows to check the stability of obtained solution with respect to white noise with zero average and no correlations in time and space, *i.e*., 〈*χ*(**r**_*i*_, *t*_*j*_)〉 = 0, 〈*χ*(**r**_*i*_, *t*_*j*_)*χ*(**r**_*k*_, *t*_*l*_)〉 = *χ*^2^*δ*_*i*,*k*_*δ*_*jl*_, with the noise intensity *χ* and the Kronecker delta *δ* calculated in discrete grid points (see ref.^81^). Most numerical calculations were done without noise (*χ* = 0), except the simulations where the stability of the loop against fluctuations was checked in Supplementary Note 5.

Josephson tunneling between the layers is incorporated using the following boundary conditions^36^:

2 $$\begin{array}{l}{(-i{\nabla }_{z}-{A}_{z})\psi |}_{\perp }^{bot}=\frac{i}{\mu \delta }\,[\psi (x,y,D)\,\exp \,(-i\bar{A}\delta )-\psi (x,y,d)],\\ {(-i{\nabla }_{z}-{A}_{z})\psi |}_{\perp }^{top}=\frac{i}{\mu \delta }\,[\psi (x,y,d)\,\exp \,(i\bar{A}\delta )-\psi (x,y,D)],\end{array}$$

at the interface of the junction area (excluding the pillars) with the bottom and the top superconducting layer, respectively. Here $\bar{A}$ is defined as $\bar{A}\equiv \mathrm{(1/}\delta )\,{\int }_{d}^{D}\,{A}_{z}dz$, *D* = *d* + *δ* (see Fig. 1), and *μ* is the coupling parameter (taking into account the ratio of the mass of the Cooper-pairs in the metallic and superconducting regions). The superconducting-vacuum boundary condition ${{\bf{n}}(-i\nabla -{\bf{A}})\psi |}_{n}=0$ (no supercurrent leaving the sample) is applied at the other boundaries, with **n** being the unit vector normal to the surface. Equation (1) is coupled with the equation of the current continuity which can be rewritten as an equation for the electrostatic potential *φ*: $\nabla (\frac{{\sigma }_{n}({\bf{r}})}{{\sigma }_{n}}\nabla \phi )={\rm{div}}({{\bf{j}}}_{{\bf{s}}})$, where *σ*_*n*_ is the normal-state conductivity of the used superconductor (*σ*_*n*_(**r**) can be taken lower inside the junction, as ${\sigma }_{n}^{J}/{\sigma }_{n}=\zeta  < 1$), and the superconducting current component is given by

3 $${{\bf{j}}}_{{\bf{s}}}=\frac{1}{2i}\,({\psi }^{\ast }\nabla \psi -\psi \nabla {\psi }^{\ast })-{\bf{A}}|\psi {|}^{2}.$$

From Eq. (2), we derive the Josephson current across the junction as^36^

4 $$\begin{array}{rcl}{j}_{s\perp }(={j}_{J}) & = & [\psi (x,y,D)\,\exp \,(-i\bar{A}\delta )\,{\psi }^{\ast }(x,y,d)\\  &  & -{\psi }^{\ast }(x,y,D)\,\exp \,(i\bar{A}\delta )\,\psi (x,y,d\mathrm{)]/2}i\mu \delta .\end{array}$$

Using Eq. (4) as well as relations *ψ*(*x*, *y*, *D*) = |*ψ*_*top*_|exp(*iθ*_*top*_) and *ψ*(*x*, *y*, *d*) = |*ψ*_*bot*_|exp(*iθ*_*bot*_), one can easily derive the usual expression for the Josephson current

5 $${j}_{J}={j}_{c}\,\sin ({\theta }_{top}-{\theta }_{bot}-\bar{A}\delta )$$

in the junction between two superconducting layers, with *j*_*c*_ = |*ψ*_*top*_*ψ*_*bot*_|/*μδ*. Near pillars, the value |*ψ*_*top*_*ψ*_*bot*_| becomes a strong function of (*x*, *y*), resulting in a significant deviation of the current from the well-known Josephson dependence of the superconducting current in a Josephson junction where *j*_*c*_ is constant. For spatially uniform critical current *j*_*c*_, the Eq. (5) is linked to the usual Josephson energy term ∝ ${E}_{J}\,\cos ({\theta }_{top}-{\theta }_{bot}-\bar{A}\delta )$ with *E*_*J*_ $\propto$ *j*_*c*_, hence the current between top and bottom superconducting layer in our system (away from pillars) exactly corresponds to the usual Josephson current accumulating the Josephson energy in the Josephson junction between top and bottom superconducting layers. Note that the total free energy ${\mathcal F}$ of the considered system contains both GL energy of the superconducting region (both layers and pillars) as well as the Josephson coupling energy of the junction:

6 $$\begin{array}{rcl} {\mathcal F}  & = & {\int }_{{V}^{\ast }}\,dxdydz\{|(\nabla -i{\bf{A}})\psi {|}^{2}-f({\bf{r}})|\psi {|}^{2}+\frac{1}{2}|\psi {|}^{4}\}\\  &  & +\frac{1}{\mu \delta }\,{\int }_{{S}^{\ast }}\,dxdy{|\psi (x,y,D)\exp (-i\bar{A}\delta )-\psi (x,y,d)|}^{2}\\  &  & +\frac{{H}_{c2}^{2}}{2{H}_{c}^{2}}\,{\int }_{V}\,dxdydz{(\nabla \times {\bf{A}})}^{2},\end{array}$$

where the total free energy ${\mathcal F}$ is normalized by ${H}_{c}^{2}\mathrm{/4}\pi$ (*H*_*c*_ being the thermodynamic critical field). The integration region V^*^ contains both superconducting layers as well as all pillars, while integration of the magnetic energy is performed over entire space. Josephson coupling in ${\mathcal F}$ is represented by the integral over two-dimensional surface S^*^ which covers the bottom surface of the top layer and the top side of the bottom layer, except of pillar areas. By calculating functional derivative of this free energy with respect to superconducting order parameter ψ^*^ and vector potential **A**, we can easily derive equations (1) and (3) as well as the Josephson relation (2), which we used in our simulations.

The external current is injected through the normal-metal leads, simulated by *ψ* = 0 and the boundary condition for the electrostatic potential $\nabla \phi =\pm j$, with *j* = *I*/*S* being the applied current density, with *I* the applied current and *S* the area of the top surface of the sample. In all equations, the length is expressed in units of the coherence length *ξ* and the vector potential is scaled to *ϕ*_0_/(2*πξ*) (where *ϕ*_0_ is the magnetic flux quantum), so that the unit for magnetic field is *H*_*c*2_ = *ϕ*_0_/(2*πξ*^2^). Time is in units of *t*_0_ = 4*πλξσ*_*n*_/*c*^2^, the electrostatic potential is in units of *V*_0_ = *cϕ*_0_/8*π*^2^*σ*_*n*_*λ*^2^, and the current density is scaled to *j*_0_ = *cϕ*_0_/8*π*^2^*λ*^2^*ξ*. The parameters *u* was taken as 5.79 (as stemming from microscopic theory, see ref.^42^), whereas *μ* and normal-state conductivity of the junction were varied in a broader range: 1 < *μ* < 20 and 0.01 < *ζ* < 1. Using *ξ*(0) = 10 nm, *λ*(0) = 200 nm, critical temperature *T*_*c*_ = 7 K, and *ρ*_*n*_ = 1/*σ*_*n*_ = 18.7 *μ*Ωcm, which are reasonable values for Nb thin films^82^, and taking typical experimental temperature of 4.2 K, one obtains *t*_0_ ≈ 0.67 ps, *j*_0_ ≈ 1.66 · 10^7^ A/cm^2^, and *V*_0_ ≈ 0.49 mV. Therefore, we report observation of Josephson loops in a rather broad range of junction parameters: the critical Josephson current density 5 × 10^4^ < *j*_*c*_ < 10^6^ A/cm^2^, and the sample resistance-area product in the interval 0.03–0.3 Ω*μ*m^2^. We neglected demagnetization effects, which is valid for extreme type-II superconductors. The used coupled nonlinear differential equations were discretized using the link variable approach (see, for example, refs^83.84^) and solved self-consistently in three dimensions using explicit Euler (for *ψ*) and multigrid (for *φ*) iterative procedures.

We also ensure that the neglected magnetic field generated by the current in the sample does not affect the predicted effects. This has been confirmed by using the iterational procedure: first, calculating the distribution of the order parameter and currents inside the sample in the spatially homogeneous field equal to the applied field, and, then, using the 3D Maxwell equations with the obtained current density to calculate corrections to the magnetic field distribution, and consequently the order parameter as well^85^. After such self-consistent solution of both Ginzburg-Landau and Maxwell equations, the obtained results indicate that the corrections to the magnetic field are negligible (see Supplementary Note 1).

## Electronic supplementary material

Formation of Josephson loops around the barriers in the junction through vortex cutting and recombination

Change of chirality of the Josephson loop prior to annihilation with a moving Josephson vortex: the Cooper-pair density evolution

Change of chirality of the Josephson loop prior to annihilation with a moving Josephson vortex: evolution of the phase of the order parameter

Supplementary Material

## References

[1] Dennis MR, King RP, Jack B, O’Holleran K, Padgett M (2010). Isolated optical vortex knots. Nature Phys..

[2] Irvine WTM, Bouwmeester D (2008). Linked and knotted beams of light. Nature Phys..

[3] Desyatnikov AS, Buccoliero D, Dennis MR, Kivshar Y (2012). Spontaneous knotting of self-trapped waves. Scientific Reports.

[4] Berger MA (1999). Introduction to magnetic helicity. Plasma Phys. Control. Fusion.

[5] Lapointe C, Mason T, Smalyukh II (2009). Shape-controlled colloidal interactions in nematic liquid crystals. Science.

[6] Smalyukh II, Lansac Y, Clark NA, Trivedi RP (2010). Three-dimensional structure and multistable optical switching of triple-twisted particle-like excitations in anisotropic fluids. Nature Mater..

[7] Tkalec U, Ravnik M, Copar S, Zumer S, Musevic I (2011). Reconfigurable knots and links in chiral nematic colloids. Science.

[8] Alexander GP, Chen BG, Matsumoto EA, Kamien RD (2012). Disclination Loops, Point Defects, and All That in Nematic Liquid Crystals. Rev. Mod. Phys..

[9] Kawaguchi Y, Nitta M, Ueda U (2008). Knots in a Spinor Bose-Einstein Condensate. Phys. Rev. Lett..

[10] Bulgac A, Luo YL, Magierski P, Roche KJ, Yu J (2011). Real-Time Dynamics of Quantized Vortices in a Unitary Fermi Superfluid. Science.

[11] Kleckner D, Irvine WTM (2013). Creation and dynamics of knotted vortices. Nature Phys..

[12] Moffatt HK (1981). Some developments in the theory of turbulence. J. Fluid Mech..

[13] Ricca RL, Berger MA (1996). Topological ideas and fluid mechanics. Phys. Today.

[14] Blatter G, Geshkenbein VB, Larkin AI, Vinokur VM (1994). Vortices in high-temperature superconductors. Rev. Mod. Phys..

[15] Lee PA, Nagaosa N, Wen X-G (2006). Doping a Mott insulator: Physics of high-temperature superconductivity. Rev. Mod. Phys..

[16] Olsson P, Teitel S (2003). Search for a vortex loop blowout transition in a type-II superconductor in a finite magnetic field. Phys. Rev. B.

[17] Carneiro G (1992). Influence of vortex-loop fluctuations on equilibrium properties of layered superconductors. I. Mean-field approach. Phys. Rev. B.

[18] Chattopadhyay B, Shenoy SR (1994). Kosterlitz-Thouless signatures from 3D vortex loops in layered superconductors. Phys. Rev. Lett..

[19] Nelson DR (1988). Vortex Entanglement in High-*T*_*c*_ Superconductors. Phys. Rev. Lett..

[20] Olson Reichhardt CJ, Hastings MB (2004). Do Vortices Entangle?. Phys. Rev. Lett..

[21] Schonenberger A, Larkin A, Heeb E, Geshkenbein V, Blatter G (1996). Strong Pinning and Plastic Deformations of the Vortex Lattice. Phys. Rev. Lett..

[22] Samokhvalov AV (1996). Vortex loops entry into type-II superconductors. Physica C.

[23] Doria MM, Romaguera AR, de C, Milošević MV, Peeters FM (2007). Threefold onset of vortex loops in superconductors with a magnetic core. Europhys. Lett..

[24] Brandt EH (1980). Continuous vortex cutting in type II superconductors with longitudinal current. J. Low Temp. Phys..

[25] Marsh GE (1994). Flux flow and flux cutting in type-II superconductors carrying a longitudinal current. Phys. Rev. B.

[26] Berdiyorov GR (2013). Current-induced cutting and recombination of magnetic superconducting vortex loops in mesoscopic superconductor-ferromagnet heterostructures. Phys. Rev. B.

[27] Palau A, Dinner R, Durrell JH, Blamire MG (2008). Vortex Breaking and Cutting in Type II Superconductors. Phys. Rev. Lett..

[28] Cubitt R, Campbell AS, Forgan EM, Dewhurst CD, Yang G (2009). Investigation of vortex structures in a current-carrying Nb wire. Supercond. Sci. Technol..

[29] Franz A, Wallraff A, Ustinov AV (2001). Magnetic field penetration in a long Josephson junction imbedded in a wide stripline. J. Appl. Phys..

[30] Fistul MV (2003). Quantum Dissociation of a Vortex-Antivortex Pair in a Long Josephson Junction. Phys. Rev. Lett..

[31] Fistul MV, Ustinov AV (2003). Josephson vortex interaction mediated by cavity modes: Tunable coupling for superconducting qubits. Phys. Rev. B.

[32] Nappi C, Lisitskiy MP, Rotoli G, Cristiano R, Barone A (2004). New fluxon resonant mechanism in annular Josephson tunnel structures. Phys. Rev. Lett..

[33] Savel’ev S, Yampol’skii VA, Rakhmanov AL, Nori F (2010). Terahertz Josephson plasma waves in layered superconductors: spectrum generation nonlinear and quantum phenomena. Rep. Prog. Phys..

[34] Roditchev D (2015). Direct observation of Josephson vortex cores. Nature Physics.

[35] Berdiyorov, G. R., Milošević, M. V., Savel’ev, S., Kusmartsev, F. & Peeters, F. M. Parametric amplification of vortex-antivortex pair generation in a Josephson junction. *Phys. Rev. B* **90**, 134505 (2014).

[36] Berdiyorov GR, Savel’ev S, Milošević MV, Kusmartsev F, Peeters FM (2013). Synchronized dynamics of Josephson vortices in artificial stacks of SNS Josephson junctions under both dc and ac bias currents. Phys. Rev. B.

[37] Liu C-Y, Berdiyorov GR, Milošević MV (2011). Vortex states in layered mesoscopic superconductors. Phys. Rev. B.

[38] Amundsen M, Linder J (2016). General solution of 2D and 3D superconducting quasiclassical systems: coalescing vortices and nanoisland geometries. Scientific Reports.

[39] Patel V, Lukens JE (2000). Self-Shunted Nb/AIOx/Nb Josephson Junctions. IEEE Trans. Appl. Supercond..

[40] Meckbach JM (2013). Sub-*μ*m Josephson Junctions for Superconducting Quantum Devices. IEEE Trans. Appl. Supercond..

[41] Lombardo, J. *et al*. *In situ* tailoring of superconducting junctions via electro-annealing. *Nanoscale* **10**, 1987 (2018). 10.1039/c7nr08571k 29319073

[42] Kopnin, N. *Theory of Nonequilibrium Superconductivity* (Oxford University Press, 2001).

[43] Embon L (2017). Imaging of super-fast dynamics and flow instabilities of superconducting vortices. Nature Commun..

[44] Takenaka K, Mizuhashi K, Takagi H, Uchida S (1994). Interplane charge transport in YBa_2_Cu_3_O_7*y*_: Spin-gap effect on in-plane and out-of-plane resistivity. Phys. Rev. B.

[45] Koshelev AE (1999). Crossing Lattices, Vortex Chains, and Angular Dependence of Melting Line in Layered Superconductors. Phys. Rev. Lett..

[46] Savel’ev S, Nori F (2002). Experimentally realizable devices for controlling the motion of magnetic flux quanta in anisotropic superconductors. Nature Mater..

[47] D’Anna G (2002). Controlling the motion of quanta. Nature Mater..

[48] Cole D (2006). Ratchet without spatial asymmetry: Controlling the motion of magnetic flux quanta using time-asymmetric drives. Nature Mater..

[49] Tonomura A (2006). Superconductivity: Conveyor belts for magnetic flux quanta. Nature Mater.

[50] Cole D, Bending SJ, Savel’ev S, Tamegai T, Nori F (2006). Manipulation of magnetic-flux landscapes in superconducting Bi_2_Sr_2_CaCu_2_O_8+*δ*_ crystals. Europhys. Lett..

[51] Cole D (2006). Vortex pumps in the crossing lattices regime of highly anisotropic layered superconductors. Physica C.

[52] Bending SJ, Cole D, Savel’ev S, Nori F, Tamegai T (2007). Ratchet without spatial asymmetry: Controlling the motion of magnetic flux quanta using time-asymmetric drives. Physica C.

[53] Grigorenko A, Bending S, Tamegai T, Ooi S, Henini M (2001). A one-dimensional chain state of vortex matter. Nature (London).

[54] Kirtley JR (2010). Fundamental studies of superconductors using scanning magnetic imaging. Rep. Prog. Phys..

[55] Keay JC (2009). Sequential vortex hopping in an array of artificial pinning centers. Phys. Rev. B.

[56] Guillamón I (2009). Direct observation of melting in a two-dimensional superconducting vortex lattice. Nature Phys..

[57] Cren T, Serrier-Garcia L, Debontridder F, Roditchev D (2011). Vortex Fusion and Giant Vortex States in Confined Superconducting Condensates. Phys. Rev. Lett..

[58] Vlasko-Vlasov VK, Koshelev A, Welp U, Crabtree GW, Kadowaki K (2002). Decoration of Josephson vortices by pancake vortices in Bi_2_Sr_2_CaCu_2_O_8+*d*_. Phys. Rev. B.

[59] Tokunaga M, Tamegai T, Fasano Y, de la Cruz F (2003). Direct observations of the vortex chain state in Bi_2_Sr_2_CaCu_2_O_8+*y*_ by Bitter decoration. Phys. Rev. B.

[60] Grigorenko AN (2005). Tilt of Pancake Vortex Stacks in Layered Superconductors in the Crossing Lattice Regime. Phys. Rev. Lett..

[61] Koshelev AE, Latyshev YuI, Konczykowski M (2006). Slowing down the Josephson vortex lattice in Bi_2_Sr_2_CaCu_2_O_8+*δ*_ with pancake vortices. Phys. Rev. B.

[62] Crisan A, Bending SJ, Tamegai T (2008). Manipulation of pancake vortices by rotating a Josephson vortex lattice. Supercond. Sci. Technol..

[63] Segev Y (2011). Lamellar Solid-Liquid Mesophase Nucleated by Josephson Vortices at the Melting of the Vortex Lattice in Bi_2_Sr_2_CaCu_2_O_8+*δ*_ Superconductor. Phys. Rev. Lett..

[64] Landau IL, Keller H (2007). On the interpretation of muon-spin-rotation experiments in the mixed state of type-II superconductors. Physica C.

[65] Brandt EH (2009). Muon spin rotation and the vortex lattice in superconductors. Physica B.

[66] Abraimov D (2004). Scanning laser imaging of dissipation in YBa_2_Cu_3_O_7−*δ*_ coated conductors. Appl. Phys. Lett..

[67] Jelić ŽL, Milošević MV, Silhanek AV (2016). Velocimetry of superconducting vortices based on stroboscopic resonances. Sci. Rep..

[68] Silhanek AV (2010). Formation of stripelike flux patterns obtained by freezing kinematic vortices in a superconducting Pb film. Phys. Rev. Lett..

[69] Klein O (1929). Die Reflexion von Elektronen an einem Potentialsprung nach der relativistischen Dynamik von Dirac. Zeitschrift für Physik.

[70] Savel’ev S, Yampol’skii VA, Rakhmanov AL, Nori F (2005). Generation of tunable terahertz out-of-plane radiation using Josephson vortices in modulated layered superconductors. Phys. Rev. B.

[71] Savel’ev S, Yampol’skii VA, Rakhmanov AL, Nori F (2006). Generation of tunable terahertz radiation using Josephson vortices: Transition and Cherenkov radiation. Physica C.

[72] Savel’ev S, Rakhmanov AL, Nori F (2005). Using Josephson Vortex Lattices to Control Terahertz Radiation: Tunable Transparency and Terahertz Photonic Crystals. Phys. Rev. Lett..

[73] Savel’ev S, Rakhmanov AL, Nori F (2006). Josephson vortex lattices as scatterers of terahertz radiation: Giant magneto-optical effect and Doppler effect using terahertz tunable photonic crystals. Phys. Rev. B.

[74] Savel’ev S, Yampol’skii VA, Nori F (2005). Surface Josephson plasma waves in layered superconductors. Phys. Rev. Lett..

[75] Savel’ev S, Rakhmanov AL, Yampol’skii VA, Nori F (2006). Analogues of nonlinear optics using terahertz Josephson plasma waves in layered superconductors. Nature Phys..

[76] Savel’ev S, Rakhmanov AL, Nori F (2007). Quantum terahertz electrodynamics and macroscopic quantum tunneling in layered superconductors. Phys. Rev. Lett..

[77] Savel’ev S, Yampol’skii VA, Rakhmanov AL, Nori F (2007). Layered superconductors as nonlinear waveguides for terahertz waves. Phys. Rev. B.

[78] Kusmartsev FV (1992). Destruction of Meissner effect in granular high-temperature superconductors. Phys. Rev. Lett..

[79] Braunisch W (1992). Paramagnetic Meissner effect in Bi high-temperature superconductors. Phys. Rev. Lett..

[80] Donelly RJ (1991). Quantized Vortices in Helium II.

[81] Vodolazov DY, Peeters FM (2002). Dynamic transitions between metastable states in a superconducting ring. Phys. Rev. B.

[82] Gubin AI, Il’in KS, Vitusevich SA, Siegel M, Klein N (2005). Dependence of magnetic penetration depth on the thickness of superconducting Nb thin films. Phys. Rev. B.

[83] Kato R, Enomoto Y, Maekawa S (1993). Effects of the surface boundary on the magnetization process in type-II superconductors. Phys. Rev. B.

[84] Milošević MV, Geurts R (2010). The Ginzburg-Landau theory in application. Physica C.

[85] Berdiyorov GR, Hernandez AD, Peeters FM (2009). Confinement Effects on Intermediate-State Flux Patterns in Mesoscopic Type-I Superconductors. Phys. Rev. Lett..

