# Supplementary material for: Josephson vortex loops in nanostructured Josephson junctions
Source: Sci Rep. 2018 Feb 9;8:2733. doi: 10.1038/s41598-018-21015-7 (PMC5807441; doi:10.1038/s41598-018-21015-7)
Supplement: Supplementary file 4 — Supplementary Material [file 41598_2018_21015_MOESM4_ESM.pdf]

Supplementary Material, article *Josephson vortex loops in nanostructured Josephson junctions*

G. R. Berdiyorov, M. V. Milošević, F. Kusmartsev, F. M. Peeters, and S. Savel'ev
(Dated: January 11, 2018)

Supplementary Figures

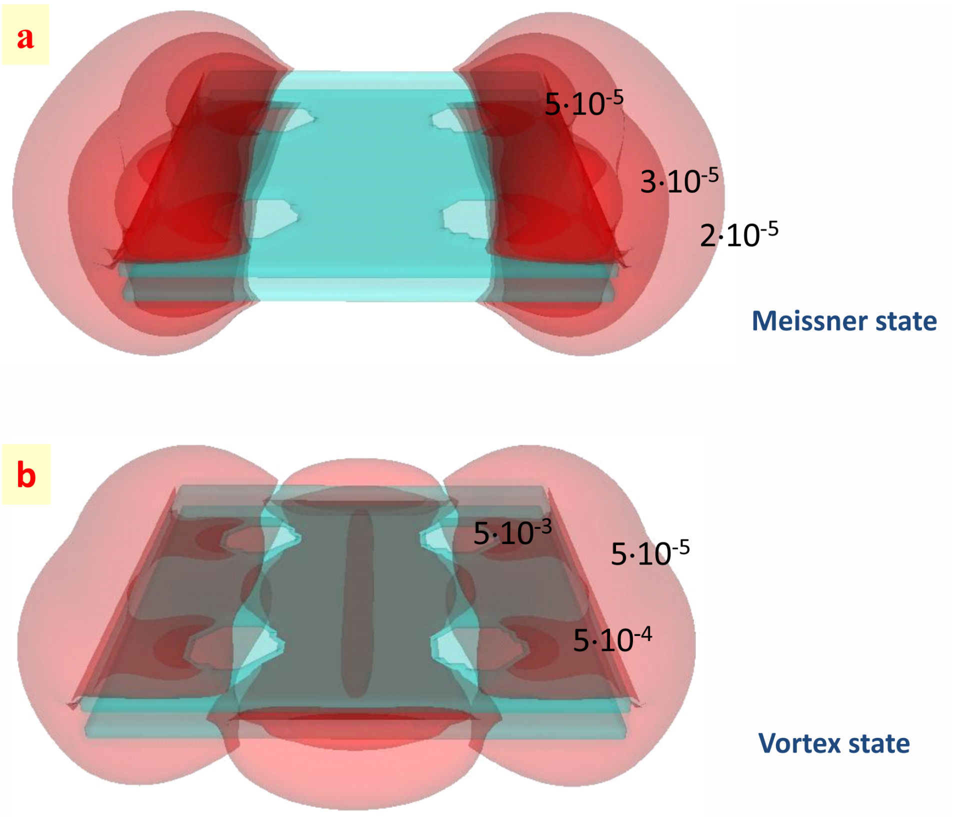

FIG. Supplementary Figure 1: **Magnetic response of the considered samples.** Isosurface plots of the magnetic field distribution [$H_m = (H_{tot} - H)/H$, where H_{tot} is the total local magnetic field and H is the applied magnetic field] around the sample of Fig. 2 of the manuscript, for Nb values of λ and ξ ($\lambda/\xi \approx 20$). (a) The system is in the Meissner state, thus vortex-free, at applied field $H = 0.005H_{c2}$. (b) The same as in (a), but for the system in the vortex state (one straight Josephson vortex, at applied field $H = 0.01H_{c2}$).

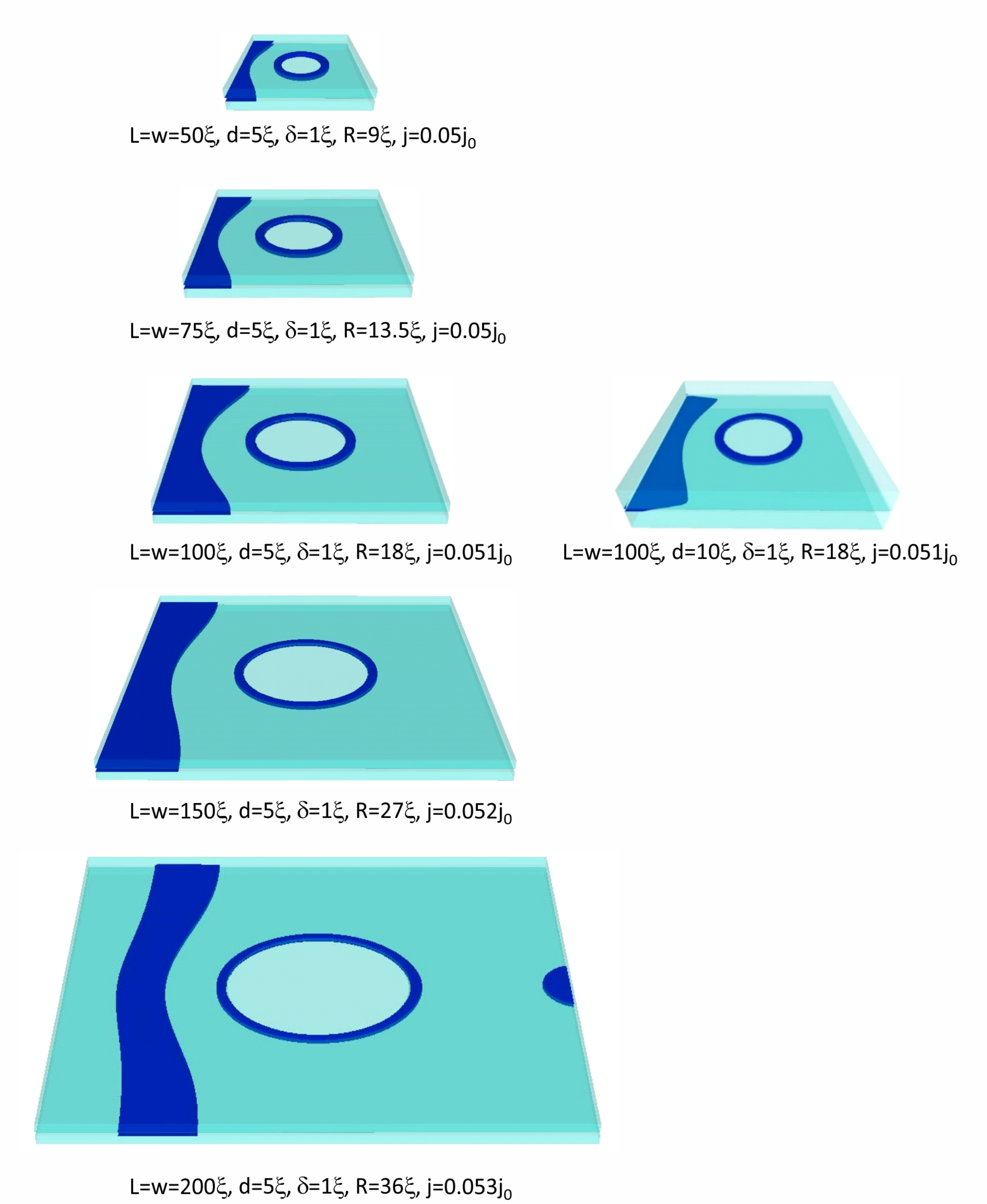

FIG. Supplementary Figure 2: **Possible size of Josephson loops.** Isosurface plots of the Cooper-pair density (taken isovalue is 30% of $|\psi|^2_{max}$, such that dark blue color outlines vortices), in samples with indicated parameters, illustrating stable Josephson vortex loops even around pillars of very large radius compared to coherence length ξ.

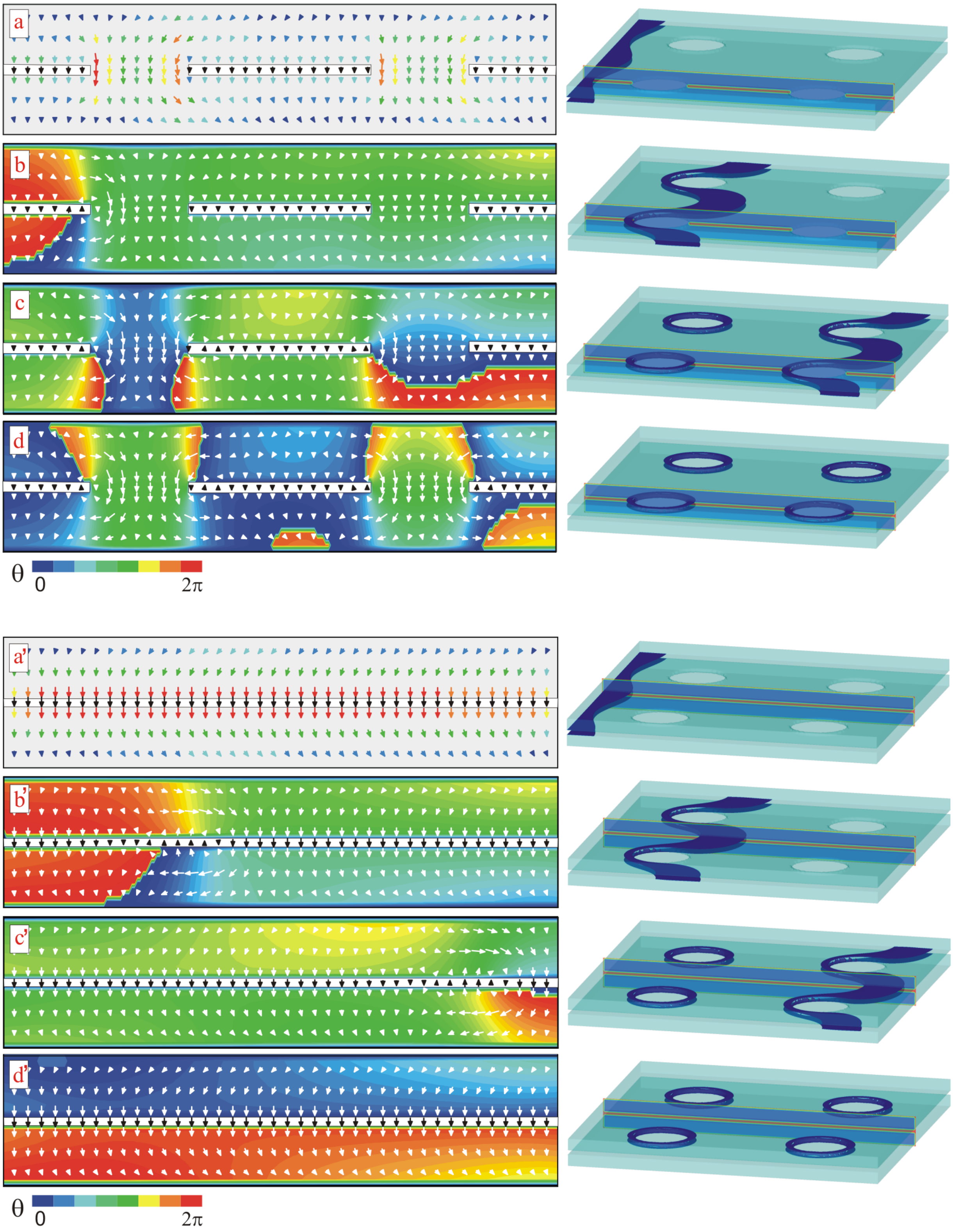

FIG. Supplementary Figure 3: **Current distribution in the sample during vortex motion.** $a-d$ Distribution of supercurrents inside the junction and the pillars, for several characteristic states formed in the applied magnetic field and current (corresponding to Fig. 2 of the manuscript), in the cross-section shown in the isosurface plots of the Cooper-pair density on the right (taken isovalue is 30% of $|\psi|^2_{max}$, such that dark blue color outlines vortices). In panel a, colors visually emphasize the magnitude of the supercurrent density (the Josephson current density is shown in black in all panels). In panels $b-d$ the supercurrent distribution is superimposed onto the contourplot of the phase of the order parameter, for better visibility of the vortex singularities. $a'-d'$ The same as in $a-d$, respectively, but for a cross-sectional plane away from the pillars (see panels on the right). Note that the redistribution of the current with the pillar (a-d) is of the same order as in the plain junction (a'-d') with Josephson vortices indicating that the loop formation does not result in any extraordinary gradients of the current density and the order parameter.

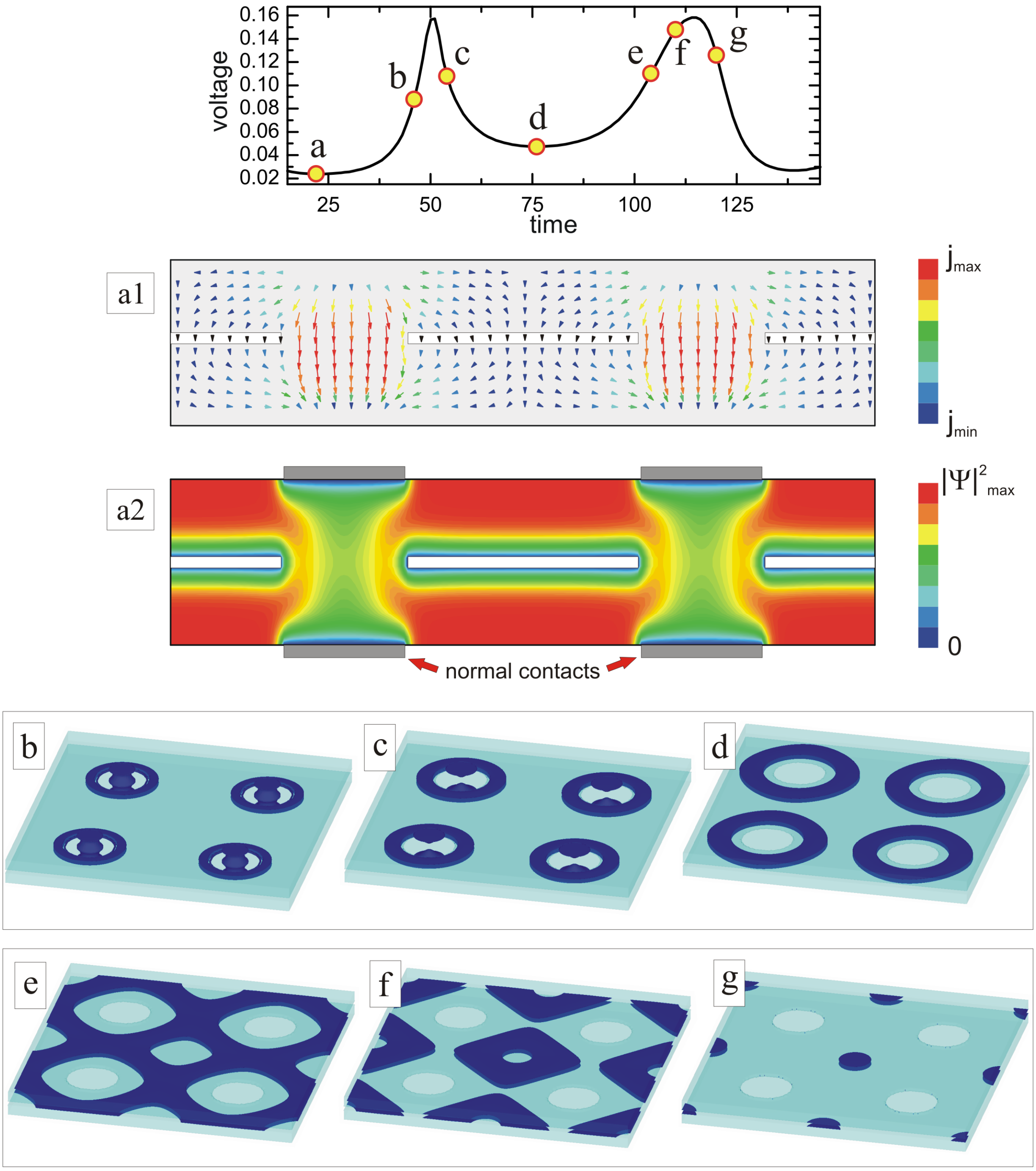

FIG. Supplementary Figure 4: **Josephson vortex loops created by locally injected current.** Top panel: A $V(t)$ curve of the sample with $L = 100\xi$, $w = 100\xi$, $d = 5\xi$, $\delta = 1\xi$, and $R = 9\xi$, for an applied current density $j = 0.28 j_0$ injected through the circular metallic contacts attached to the top and the bottom surface of the device so that the current flows mainly through the pillars, in the absence of applied magnetic field. Panel $a1$: Supercurrent distribution inside the sample at $t = 24t_0$ (the current is switched on at $t = 0$). Panel $a2$: Contourplot of the Cooper-pair density inside the sample at $t = 24t_0$. Panels $b - g$: Isosurface plots of the Cooper-pair density at other times indicated on the $V(t)$ curve (taken isovalue is 30% of $|\psi|^2_{max}$, such that dark blue color outlines vortices).

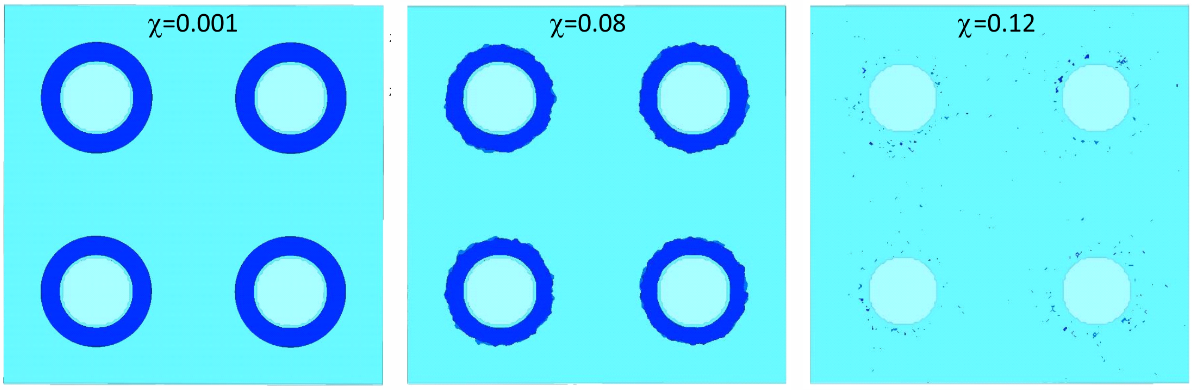

FIG. Supplementary Figure 5: **Effect of fluctuations on Josephson loops.** Isosurface plots of the Cooper-pair density (taken isovalue is 30% of $|\psi|^2_{max}$, such that dark blue color outlines vortices), showing stability of the Josephson loops in a sample corresponding to Fig.2i of the main manuscript, after magnetic field and current are removed, but in presence of fluctuations uncorrelated in space and time, of magnitude χ (normalized to bulk superconducting order parameter).

Supplementary Notes

Supplementary Note 1: Demagnetization effects

In our simulations, the magnetic response of the superconducting sample was neglected. This is known to be justified for thin films, especially those of extreme type-II superconductors (with large Ginzburg-Landau (GL) parameter $\kappa = \lambda/\xi$). Since Josephson junctions are typically made of such superconductors (e.g. Nb-based ones, where $\kappa \approx 20$, or YBCO, with $\kappa > 100$), our assumption of negligible contribution of the screening currents to the magnetic field is justified. Nevertheless, for the completeness of the study, we show in Supplementary Figure 1 the inhomogeneous distribution of the stray magnetic field due to the screening currents of the sample corresponding to Fig. 2 of the main manuscript, in two cases: a) when sample is free of vortices, and b) when one Josephson vortex is present in the sample. In either case, the screening field is far smaller than the applied magnetic field, and can be safely neglected in the analysis of emergent vortex matter.

The magnetic field shown in Supplementary Figure 1 is calculated within the same model, selfconsistently with the added second GL equation for the magnetic field due to superconducting currents, as detailed in Ref. 65 of the main manuscript. In this iterative approach, after initial calculation of the spatial distribution of the order parameter based on just external magnetic field, the supercurrent and its stray magnetic field are calculated in whole space and fed back to the equation for the order parameter. Typically, after $10^5 - 10^6$ such iterations the convergence is reached for both the order parameter and the magnetic field in the 3D space.

Supplementary Note 2: How large can Josephson loops be?

In this note, we reiterate the important point of the main manuscript - Josephson loops are not only a new and table topological object in superconductivity, they can be made rather large compared to other known vortical objects. As shown in Supplementary Figure 2, the loops can be created around larger pillars, so that their radius follows that of a pillar. Here we make use of large elasticity of a moving Josephson vortex, due to the absence of its core, and thereby facilitated flexibility of the vortex line around an obstacle in its path. In the example shown in Supplementary Figure 2 we have stabilized Josephson loops with size up to a micrometer, assuming Nb as the material for the sample. For large samples, gradients of both the order parameter and current density become very modest, further supporting the validity of our theoretical approach. We have deliberately also considered thicker films, to demonstrate clearly that 3D effects and somewhat different distribution of the current do not affect our main claims.

Supplementary Note 3: Effect of pillars on the current distribution

In our simulations, the current was injected uniformly through the top surface of the upper superconducting layer and the same current was uniformly taken out at the bottom surface of the bottom superconducting layer. However, the current distribution in the system changes considerably in the presence of pillars, made of same or different superconducting material, and our three-dimensional calculations fully account for this fact. To illustrate this, complementary to the *planar* current density plots in Fig. 5 of the main article, we show in Supplementary Figure 3 the vectorplot of the inhomogeneous current distribution in the sample corresponding to Fig. 2 of the main manuscript, for two *vertical cross-sectional planes* across the junction [across pillars (panels $a - d$) and away from pillars (panels $a' - d'$)], for selected characteristic vortex states. This clearly demonstrates that we always consider full 3D distribution of the current across the Josephson junction in the simulations, and that effects of such current distribution are accounted for in our results.

Supplementary Note 4: Josephson vortex loops created by strong localized current

We have already pointed out that uniformly applied current redistributes on the junction and the current density is always larger inside the pillars compared to the rest of the junction. Hence one wonders to which extent this larger current density inside the pillars can affect the mechanism of loop formation. To reveal this effect, we have conducted simulations where current was applied locally at the top superconducting layer directly above the pillars [such that contacts are attached only on the pillars, not on the entire plane of the junction], and otherwise same parameters as in Fig. 2 of the main article. The current is then mostly focused through the pillars (see panel $a1$ in Supplementary Figure 4), with consequently suppressed superconductivity there (panel $a2$ in Supplementary Figure 4). The increased current density will eventually lead to the nucleation of vortex loops from within each pillar, as shown in panel b of Supplementary Figure 4. With time, these loops expand under the action of the Lorentz force (panels c and d) until they reach each other and/or sample boundaries (panel e). Subsequently, vortex loops around the pillars recombine into vortex loops between the pillars (panel f), with different chirality, and more importantly, with nothing to prevent their shrinkage and collapse under further action of the Lorentz force (panel g). Thus, vortex loops can be created by the supercurrent guided mainly through the pillars, and this is a completely different scenario from our original proposal. However, for that one would need spatially nanoengineered current leads, very large applied current density (in the shown example, more than 5 times larger compared to the original proposal), and carefully

chosen parameters of the pillars so that superconductivity there is not fully destroyed by the current (otherwise, there is nothing remaining to stabilize loops if current is switched off, plus thermal quench due to Joule heating is very likely).

Supplementary Note 5: Effect of fluctuations

In our simulations, possible fluctuations of the superconducting order parameter were not taken into account. In principle, our results are given in dimensionless units, and are applicable even at low temperatures where thermal fluctuations are not relevant. Still, to address the possible detrimental effects of fluctuations on stability of Josephson vortex loops, we have repeated the calculations related to Fig.2 of the main manuscript, with white noise $\chi(\mathbf{r}, t)$ added to the first Ginzburg-Landau equation (the numerical method described in Ref. 63 of the main text). In this manner, the amplitude of χ directly translates into the superconducting order parameter ψ. In Supplementary Figure 5, we directly show that Josephson vortex loops become unstable only when magnitude of fluctuations exceeds 10% of the bulk order parameter of the considered sample. At the same time, the threshold values of applied current to induce moving vortices in the system, and create Josephson loops, are nearly unaffected by the fluctuations (in the regime of weak-to-moderate fluctuations).
